# A Locally Both Leptokurtic and Fat-Tailed Distribution with Application in a Bayesian Stochastic Volatility Model

**DOI:** 10.3390/e23060689

**Published:** 2021-05-30

**Authors:** Łukasz Lenart, Anna Pajor, Łukasz Kwiatkowski

**Affiliations:** 1 Department of Mathematics, Cracow University of Economics, ul. Rakowicka 27, 31-510 Kraków, Poland; pajora@uek.krakow.pl or; 2 Department of Financial Mathematics, Jagiellonian University in Kraków, ul. Prof. Stanisława Łojasiewicza 6, 30-348 Kraków, Poland; 3 Department of Econometrics and Operations Research, Cracow University of Economics, ul. Rakowicka 27, 31-510 Kraków, Poland; kwiatkol@uek.krakow.pl

**Keywords:** stochastic volatility, Markov chain Monte Carlo, Bayesian inference, leptokurticity, heavy tails, scale mixture of normals, modelling financial data

## Abstract

In the paper, we begin with introducing a novel scale mixture of normal distribution such that its leptokurticity and fat-tailedness are only local, with this “locality” being separately controlled by two censoring parameters. This new, locally leptokurtic and fat-tailed (LLFT) distribution makes a viable alternative for other, globally leptokurtic, fat-tailed and symmetric distributions, typically entertained in financial volatility modelling. Then, we incorporate the LLFT distribution into a basic stochastic volatility (SV) model to yield a flexible alternative for common heavy-tailed SV models. For the resulting LLFT-SV model, we develop a Bayesian statistical framework and effective MCMC methods to enable posterior sampling of the parameters and latent variables. Empirical results indicate the validity of the LLFT-SV specification for modelling both “non-standard” financial time series with repeating zero returns, as well as more “typical” data on the S&P 500 and DAX indices. For the former, the LLFT-SV model is also shown to markedly outperform a common, globally heavy-tailed, t-SV alternative in terms of density forecasting. Applications of the proposed distribution in more advanced SV models seem to be easily attainable.

## 1. Introduction

Most of leptokurtic and heavy-tailed distributions, commonly entertained in financial volatility modelling, may be derived as scale mixtures of normal (SMN) distributions, with the concept dating back to [1]. SMN is a very wide and useful class of distributions, which belongs to an even wider class of elliptical distributions (see [2]). We say that a random variable ϵ follows a scale mixture of normals if it has the following stochastic representation:

(1) $$\epsilon =V^{-1/2}Z,$$

where *Z* has a standard normal distribution independent from a positive random variable *V*. If V∼Gamma(ν/2,ν/2), then ϵ follows Student’s *t*-distribution with ν degrees of freedom, while for V∼IGamma(α,β) we obtain a variance gamma distribution (see [3]). For V∼Pareto(1,ν/2) we obtain a modulated normal type I (MN type I) distribution, while V∼Beta(ν/2,1) yields a modulated normal type II distribution (see [4]), which is also known in the literature as a slash distribution (see [5]). Extensions of the latter were introduced by [6,7], who developed a modified slash (MS) and generalized modified slash (GMS) distributions, respectively.

As mentioned above, the SMN class contains many different subclasses of distributions with different tail behaviour and modal concentration in comparison with the Gaussian bell curve. In this paper, we focus particularly on two of them, namely the slash distribution, as a choice of a heavy-tailed distribution, and the MN type I distribution, featuring leptokurticity. The author of [8] shows that these two distributions are dual, meaning that the shape of the spectral density near the mode in the MN type I distribution is related to the shape of the slash distribution’s tails and vice versa.

The slash distribution seems to be one of the most common heavy-tailed distributions, with the list obviously topped by the *t*-distribution. It should be noted that as long as the slash distribution has many advantages, particularly in modelling financial data, it also suffers from some limitations, which we discuss in the following paragraph, along with some drawbacks of the MN type I distribution, stemming from its duality with the slash distribution.

Similarly to the Student’s *t*-distribution, the tails of which get thicker with the degrees of freedom approaching zero, ν→0 in the slash distribution yields the same effect (note, however, that the parameter ν in the slash distribution is not termed as degrees of freedom). This property is much appreciated in modelling heavy-tailed data, including financial returns. However, an unwanted consequence of using most of the heavy-tailed distributions (including Student’s *t*, slash, MS and GMS distributions) is that it may be the case that a relatively too large probability mass is put far in the tails. Such a shifting of the probability mass from around the mode to the tails may be somewhat unwanted (i.e., not necessarily supported by modelled data), since, informally speaking, in many (even financial) applications, there can be almost no chances of observing data points that far from the mode. To cope with such a possible discrepancy between the theoretical distribution and the data, we make some amendments to the slash distribution so that the resulting, new distribution (hereafter termed as the local slash distribution, LS) allows the tails to be freely thick but only locally up to some distance from the mode. The effect is achieved through introducing only one additional parameter, d∈(1,∞), to censor the distribution of $V^{-1/2}$ in (1) by inserting $\min\{V^{-1/2},d\}$, instead. Consequently, the tails can be thick, but only locally, while thinning out in the infinity. We show that all moments exist for this new distribution.

Apart from heavy-tailed distributions, leptokurtic ones have also found applications in many fields, including finance, with both features being typically concomitant. One of the possible choices to capture data leptokurticity is the MN type I distribution. However, it has not gained much popularity in applications, particularly financial ones, mainly due to its only thin tails. Moreover, as long as the MN type I distribution is able to capture strong a modal concentration of data, its duality to the slash distribution causes a possibly overly fat-tailedness of the latter to translate into a possibly overly high concentration of the probability mass around the mode in the former. To control for the pointedness of the MN type I distribution, in this paper, we modify it by introducing an additional, censoring parameter c∈(0,1) and supplanting the random variable $V^{-1/2}$ in (1) with $\max\{V^{-1/2},c\}$. We term the resulting construct as the local MN (LMN) type I distribution and provide a detailed description of its properties later on.

In many applications, particularly financial ones, both features of a probability distribution are desired simultaneously: the heavy tails and leptokurticity. In distributions commonly employed in practice, such as Student’s *t*, higher probability mass accumulation in the tails immediately induces higher modal concentration, with both areas of the distribution controlled for by only a single parameter. In this paper, with prior modifications of the slash and MN type I distributions, we combine the two resulting (LS and LMN type I) distributions within the SMN setting to obtain a completely new, symmetric distribution, termed as the locally both leptokurtic and fat-tailed (LLFT) distribution, which is far more flexible in modelling the outlying as well as “modal” observations. The LLFT distribution features five parameters (in its standardized form), with all moments existing but allowed to be arbitrarily large.

In this paper, we apply the proposed LLFT distribution for the error term in the basic, otherwise conditionally normal SV model that for a univariate $y_{t}$ can be written as $y_{t}=\sqrt{h_{t}}\varepsilon_{t}$, $\ln h_{t}=\gamma +\phi \left(\ln h_{t-1}-\gamma \right)+\eta_{t}$, where $\varepsilon_{t}∼N\left(0,1\right)$ and $\eta_{t}∼N\left(0,\sigma^{2}\right)$ are i.i.d. and mutually independent. Although it is well-known that such an SV model has the ability to induce leptokurtosis and heavy tails, typically observed in financial time series, its underlying conditional Gaussianity is still quite a limitation. Introducing the LLFT distribution for $\varepsilon_{t}$ aims at a more adequate capturing of the heavy tails and leptokurticity of financial assets’ returns, as compared not only to the basic but also conditionally heavy-tailed SV models, typically entertained in the literature, including t-SV and slash-SV models.

Modifications of the distribution for $\varepsilon_{t}$ has been one of the most prolific strands of SV literature (see, e.g., [9,10,11,12,13,14,15,16]). Other typical directions of generalizing the basic SV structure focus on capturing the leverage effect and asymmetry (see, e.g., [12,17,18,19]) as well as on refining the volatility process by, for example, accommodating realized volatility and long memory (see, e.g., [18,20]), or allowing for discrete, Markov switches of the parameters (see, e.g., [21,22,23]). However, we do not follow these lines of research in our current paper, focusing rather on the construction of a new distribution “from scratch” and its introduction into the basic SV model, thereby contributing to the research area of improving the conditional distribution in SV models. Extending our framework for other, more elaborate SV specifications is left for future work.

For statistical inference in the resulting LLFT-SV model, we resort to the Bayesian framework, which is typically considered for SV models (see [14,24,25,26]). We show that under certain prior structure, the marginal data density in the LLFT-SV model (and thus, the posterior distribution) is bounded even when the same values of observations repeat in the sample, which is not necessarily the case when these were to be modelled with the slash or MN type I distributions, as duly noted by [27]. The result seems essential in view of repeating (or at least strongly concentrated around some constant, e.g., the mode) values of returns that occur quite often for some individual financial assets (such as a company’s stocks).

Building on the hierarchical representation of SMN distributions, we develop an effective Markov chain Monte Carlo (MCMC) method for posterior sampling of the LLFT-SV model’s parameters and latent variables. The procedure suitably adapts standard techniques of the Metropolis–Hastings algorithm and Gibbs sampler.

To sum up, the contribution of our work is several-fold. Methodologically, a new, specific scale mixture of normals is constructed, featuring five free parameters, with two of them censoring separately the leptokurticity and fat tails to be only local, while the other three controlling for the relative weights and magnitudes of these two features in a disentangled manner. Then, we introduce this new LLFT distribution into a stochastic volatility model and develop a Bayesian framework for the resulting LLFT-SV specification, resolving some additional theoretical considerations about the existence of the posterior distribution. Next, we adapt relevant MCMC numerical methods for posterior simulations of the parameters and latent variables. Finally, we conduct an extensive empirical study of the LLFT-SV model’s workings and density forecasting performance for a selected financial asset and additionally provide a brief illustration for two common stock market indices.

The article is organized as follows. In Section 2, we define the locally both leptokurtic and fat-tailed (LLFT) scale mixture of normal distributions. In particular, we show in detail the properties of the introduced distributions, together with their relations to other, well-known distribution families. In Section 3, we introduce the stochastic volatility (SV) model incorporating the proposed distribution for the error term and develop a Bayesian framework for the resulting LLFT-SV model. Section 4 presents a real-world data analysis illustrating predictive advantages of the model. Finally, Section 5 concludes and discusses possible avenues for further research.

## 2. Introducing the Locally Both Leptokurtic and Fat-Tailed Scale Mixture of Normal Distributions

### 2.1. The Slash Distribution—A Short Review

With begin with a detailed presentation of the slash distribution’ properties, with the distribution itself parametrized according to [5], where it was introduced. Following the cited paper, we assume that

(2) $$X|W∼N(0,W^{2}),$$

where *W* has Pareto(1,ν) distribution and $N(\mu ,\sigma^{2})$ stands for the Gaussian distribution with mean μ and variance $\sigma^{2}$. Note that recently, some other parametrization has been used (see for example [14,15]), in which it is assumed that $X|\tilde{W}∼N\left(\mu ,1/\tilde{W}\right)$, where $\tilde{W}$ has $Beta(\tilde{\nu },1)$ distribution. However, both parametrizations are equivalent under $\nu =\frac{1}{2}\tilde{\nu }$, since Pareto(1,ν) is a inverse distribution for Beta(ν,1). In this paper, we choose to stick to the original parametrization by [5], as it enables a direct comparison of the slash distribution’s properties with the ones of Student’s *t*. We will discuss this later in this section. The probability density function (pdf) of the random variable *X* given by (2) is

(3) $$pdf\left(x\right)\;=\;\;\int_{1}^{\infty }\frac{W^{-\nu -1}\left(\nu e^{-\frac{x^{2}}{2W^{2}}}\right)}{\sqrt{2\pi }W}\;dW\;\;=\;\;\left\{\begin{array}{cc} \frac{2^{\frac{\nu }{2}-1}\nu {\left|x\right|}^{-\nu -1}\left(\Gamma \left(\frac{\nu +1}{2}\right)-\Gamma \left(\frac{\nu +1}{2},\frac{x^{2}}{2}\right)\right)}{\sqrt{\pi }}, & x\neq 0\\ \frac{\nu }{\sqrt{2\pi }\left(\nu +1\right)}, & x=0, \end{array}\right.$$

which is easy to obtain by noticing that the function under the integral above is the kernel of an inverse gamma distribution (under x≠0) at $W^{2}$, and Γ(a,x) denotes the upper incomplete gamma function: $\Gamma \left(a,x\right)=\int_{x}^{\infty }t^{a-1}e^{-t}dt$. The exact form of the pdf (3) of the slash distribution also admits a recurrent form, which was also shown in [5]. Density is a continuous function on the real line, symmetric and unimodal. The characteristic function of *X* given by (2) has the form $E\left(e^{itX}\right)=\frac{1}{2}\nu E_{\frac{\nu }{2}+1}\left(\frac{t^{2}}{2}\right)$, where $E_{n}\left(z\right)=\int_{1}^{\infty }\frac{e^{-tz}}{t^{n}}dt$ is the exponential integral function. It is well known that the slash distribution reduces to the standard Gaussian case as ν→∞. Note that, similarly to Student’s *t*-distribution, the moment $E(X^{2r})$ exists only under assumption ν>2r, r∈N, and is given as

(4) $$\begin{array}{c} E\left(X^{2r}\right)=E\left(\frac{\nu 2^{r}W^{2r}\Gamma \left(r+\frac{1}{2}\right)}{\sqrt{\pi }}\right)=\frac{\nu 2^{r}\Gamma \left(r+\frac{1}{2}\right)}{\sqrt{\pi }\left(\nu -2r\right)}. \end{array}$$

Hence, the variance exists only under ν>2, and is equal to $E\left(X^{2}\right)=\nu /\left(\nu -2\right)$, which is the same as for Student’s *t*-distribution with ν>2 degrees of freedom. The kurtosis of the slash distribution exists only for ν>4 and equals $Ku\left(X\right)=3{\left(\nu -2\right)}^{2}/\left[\left(\nu -4\right)\nu \right].$ For a given value of ν, the slash distribution’s kurtosis is lower than in the Student’s *t*-distribution with ν degrees of freedom, but is analogically a decreasing function of ν, tending to 3 as ν→∞, and approaching *∞* when $\nu \to 4^{+}$. Interestingly, the slash distribution features the same tail thickness as the t-distribution with ν degrees of freedom, i.e.,

$$\lim_{x\to \infty }\frac{pdf(x)\;\;\mathrm{given}\;\mathrm{by}\;(3)}{x^{-(\nu +1)}}=\frac{2^{\frac{\nu }{2}-1}\nu \Gamma \left(\frac{\nu +1}{2}\right)}{\sqrt{\pi }}\neq 0.$$

Thereby, the probability of extreme values increases as $\nu \to 0^{+}$, while for ν→∞, the slash distribution reduces to the standard Gaussian case.

In view of the above, the properties of the slash distribution with the parameter ν are comparable with the ones of Student’s *t*-distribution, with both sharing and ’suffering’ from the same restriction ensuring the existence of their $k^{th}$ moments: ν>k. Otherwise, relevant integrals do not converge since too much probability is located in the tails.

In the following subsection, we propose a modification of the slash distribution to ‘curb’ the heaviness of the tails but only far enough from the mode. In consequence, all moments of such a modified distribution exist.

### 2.2. The Concept of the Local Slash (LS) Distribution

Here, we modify the slash distribution reviewed in the previous section by introducing a censored variable in place of *W* in (2):

(5) $$X|W∼N(0,\min\left\{W^{2},d^{2}\right\}),$$

where, similarly as before, *W* follows Pareto(1,ν) distribution with ν>0, while d∈(1,∞) is a censoring parameter. The marginal distribution of *X* given by (5) depends strongly on parameter *d*, which is related to the tail thickness. The introduction of parameter *d* ensures that the tails’ thickness of *X* is upheld but only up to some distance from the mode (with the distance being controlled for by *d*), while thinning out thereafter. Since the heavy-tailedness is only “local” now, we name the new distribution as the local slash (LS) distribution.

The probability density function of the LS distribution can be easily derived and admits the form

(6) $$pdf\left(x\right)=\left\{\begin{array}{cc} \frac{\sqrt{2}d^{-\nu -1}e^{-\frac{x^{2}}{2d^{2}}}+2^{\nu /2}\nu {\left(\frac{1}{x^{2}}\right)}^{\frac{\nu +1}{2}}\left(\Gamma \left(\frac{\nu +1}{2},\frac{x^{2}}{2d^{2}}\right)-\Gamma \left(\frac{\nu +1}{2},\frac{x^{2}}{2}\right)\right)}{2\sqrt{\pi }}, & x\neq 0\\ \frac{d^{-\nu -1}\left(\nu d^{\nu +1}+1\right)}{\sqrt{2\pi }\left(\nu +1\right)} & x=0. \end{array}\right.$$

Note that the LS distribution is symmetric and is a discrete mixture of the zero-mean Gaussian distribution with variance $d^{2}$ and some unknown distribution with its pdf given by

$$\left\{\begin{array}{cc} \frac{2^{\nu /2}\nu {\left(\frac{1}{x^{2}}\right)}^{\frac{\nu +1}{2}}\left(\Gamma \left(\frac{\nu +1}{2},\frac{x^{2}}{2d^{2}}\right)-\Gamma \left(\frac{\nu +1}{2},\frac{x^{2}}{2}\right)\right)}{2\sqrt{\pi }\left(1-d^{-\nu }\right)}, & x\neq 0\\ \frac{\nu \left(d^{\nu +1}-1\right)}{\sqrt{2\pi }d\left(\nu +1\right)\left(d^{\nu }-1\right)} & x=0. \end{array}\right.$$

The weights of this mixture are $d^{-\nu }$ and $1-d^{-\nu }$, respectively. Hence, if $\nu \to 0^{+}$, then the LS distribution collapses to $N(0,d^{2})$.

The LS distribution’s cdf is given as

(7) $$cdf\left(y\right)=\left\{\begin{array}{cc} \frac{1}{2}\left(\mathrm{erf}\left(\frac{y}{\sqrt{2}}\right)+\frac{2^{\nu /2}y^{-\nu }\left(\Gamma \left(\frac{\nu +1}{2},\frac{y^{2}}{2}\right)-\Gamma \left(\frac{\nu +1}{2},\frac{y^{2}}{2d^{2}}\right)\right)}{\sqrt{\pi }}+1\right), & y\neq 0\\ \frac{1}{2}, & y=0 \end{array}\right.$$

while the characteristic function

$$E\left(e^{itX}\right)=\frac{1}{2}\left(d^{-\nu }\left(2e^{-\frac{1}{2}d^{2}t^{2}}-\nu E_{\frac{\nu }{2}+1}\left(\frac{d^{2}t^{2}}{2}\right)\right)+\nu E_{\frac{\nu }{2}+1}\left(\frac{t^{2}}{2}\right)\right).$$

One of the most important properties of the LS distribution is that the moments $E(X^{2r})$, r∈N, exist for any $\nu \in R_{+}$, and

(8) $$\begin{array}{cc} E(X^{2r}) & =E\left(E\left(X^{2r}|W\right)\right)=\left\{\begin{array}{cc} \frac{2^{r}d^{-\nu }\Gamma \left(r+\frac{1}{2}\right)\left(2rd^{2r}-\nu d^{\nu }\right)}{\sqrt{\pi }\left(2r-\nu \right)}, & 2r\neq \nu \\ \frac{2^{r}\Gamma \left(r+\frac{1}{2}\right)\left(2r\log\left(d\right)+1\right)}{\sqrt{\pi }}, & 2r=\nu , \end{array}\right. \end{array}$$

which is a continuous function of ν and *d*, for any fixed r∈N. Thus, the variance exists for any $\nu \in R_{+}$ and equals

(9) $$\begin{array}{cc} E(X^{2}) & =\left\{\begin{array}{cc} \frac{2d^{2-\nu }-\nu }{2-\nu }, & \nu \neq 2\\ 2\log(d)+1, & \nu =2. \end{array}\right. \end{array}$$

Note that for any fixed ν>0 we have

(10) $$\begin{array}{cc} \lim_{d\to \infty }E\left(X^{2}\right) & =\left\{\begin{array}{cc} \frac{\nu }{\nu -2}, & \nu >2\\ \infty , & \nu \leq 2, \end{array}\right. \end{array}$$

which means that in the limiting case of d→∞, the variance stabilizes for ν>2 and tends to the variance of the slash distribution (coinciding with the one of Student’s *t*-distribution). The above result also indicates that for any ν≤2, the variance exists and can be arbitrarily high (for sufficiently large *d*).

The kurtosis exists for any *d* and ν and equals

(11) $$\begin{array}{cc} Ku(X) & =\left\{\begin{array}{cc} \frac{3{\left(\nu -2\right)}^{2}d^{\nu }\left(\nu d^{\nu }-4d^{4}\right)}{\left(\nu -4\right){\left(\nu d^{\nu }-2d^{2}\right)}^{2}}, & \nu \neq 2,4\\ \frac{6d^{2}-3}{{\left(2\log\left(d\right)+1\right)}^{2}}, & \nu =2\\ \frac{3d^{4}\left(4\log\left(d\right)+1\right)}{{\left(1-2d^{2}\right)}^{2}}, & \nu =4. \end{array}\right. \end{array}$$

Hence,

(12) $$\begin{array}{cc} \lim_{d\to \infty }Ku\left(X\right) & =\left\{\begin{array}{cc} \frac{3{\left(\nu -2\right)}^{2}}{(\nu -4)\nu }, & \nu >4\\ \infty , & \nu \leq 4. \end{array}\right. \end{array}$$

**Figure 1:**
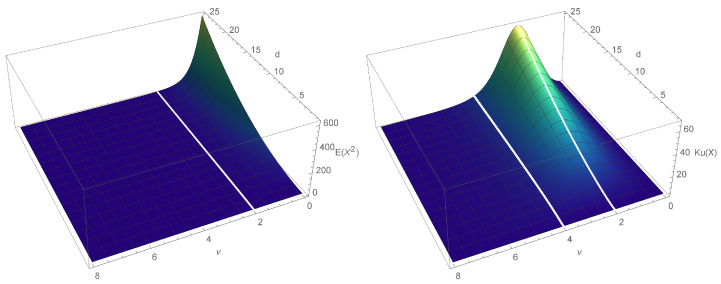
Variance (on the **left**) and kurtosis (on the **right**) for LS distribution (given by (5)) as a function of ν and *d*.

Figure 1 presents the shape of the variance and kurtosis of the LS distribution as a function of both ν and *d*. Two white lines represent the cases where ν=2 or ν=4. Note that for any fixed d>1, the variance increases as $\nu \to 0^{+}$, while the kurtosis tends to 3 with either $\nu \to 0^{+}$ or ν→∞, achieving its maximum at ν=2 (see Figure 1).

**Figure 2:**
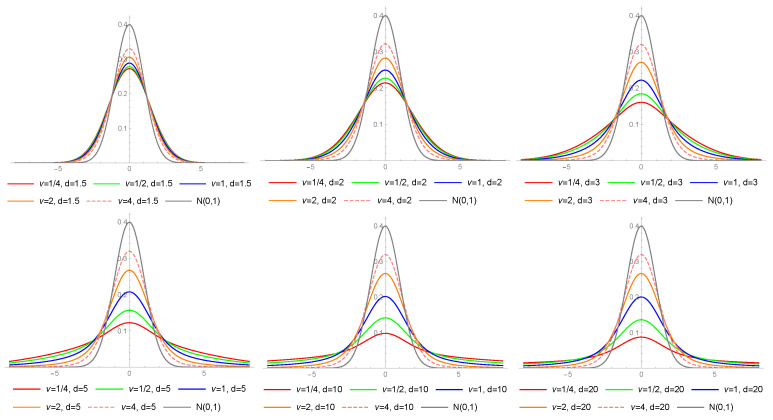
Probability density functions of the LS distribution for different values of ν and *d*.

Figure 2 presents the density functions of the LS distribution for different values of ν and *d*. Note that the higher value of *d*, the more mass is spread out on tails (for fixed ν).

### 2.3. Local Modulated Normal Type I Distribution

We begin this section with a presentation of the modulated normal (MN) type I distribution introduced by [4], which we later modify into the local modulated normal (LMN) type I distribution. Firstly, let us recall that a random variable *X* follows an MN type I distribution if

(13) $$X|R∼N(0,R^{2}),$$

where *R* is a continuous random variable on (0,1) with Beta(ρ,1) distribution, ρ>0. The pdf and cdf of random variable *X* admit the forms

(14) $$\begin{array}{cc} pdf(x) & =\int_{0}^{1}\frac{R^{\rho -1}\left(\rho e^{-\frac{x^{2}}{2R^{2}}}\right)}{\sqrt{2\pi }R}\;dR\\ \; & =\left\{\begin{array}{cc} \frac{2^{-\frac{\rho }{2}-1}\rho {\left(\frac{1}{x^{2}}\right)}^{\frac{1}{2}-\frac{\rho }{2}}\Gamma \left(\frac{1}{2}-\frac{\rho }{2},\frac{x^{2}}{2}\right)}{\sqrt{\pi }},\; & x\neq 0\\ \frac{\rho }{\sqrt{2\pi }\left(\rho -1\right)}, & x=0,\rho >1\\ \infty ,\;i.e.,\;\lim_{x\to 0}\frac{2^{-\frac{\rho }{2}-1}\rho {\left(\frac{1}{x^{2}}\right)}^{\frac{1}{2}-\frac{\rho }{2}}\Gamma \left(\frac{1}{2}-\frac{\rho }{2},\frac{x^{2}}{2}\right)}{\sqrt{\pi }}=\infty , & x=0,0<\rho \leq 1, \end{array}\right. \end{array}$$

(15) $$cdf\left(y\right)=\left\{\begin{array}{cc} -\frac{1}{2}\mathrm{erfc}\left(\frac{y}{\sqrt{2}}\right)+\frac{yE_{\frac{\rho +1}{2}}\left(\frac{y^{2}}{2}\right)}{2\sqrt{2\pi }}+1, & y\neq 0\\ \frac{1}{2}, & y=0. \end{array}\right.$$

The characteristic function equals $2^{\frac{\rho }{2}-1}\rho {\left(t^{2}\right)}^{-\frac{\rho }{2}}\left(\Gamma \left(\frac{\rho }{2}\right)-\Gamma \left(\frac{\rho }{2},\frac{t^{2}}{2}\right)\right)$ and tends to characteristic function of the standard normal distribution as ρ→∞, which indicates the equivalence of the two distributions in the limiting case. The moments exists for any ρ>0 and equals $E\left(X^{2r}\right)=\frac{\rho 2^{r}\Gamma \left(r+\frac{1}{2}\right)}{\sqrt{\pi }\left(\rho +2r\right)}$, r∈N. Hence, the variance is $E\left(X^{2}\right)=\frac{\rho }{\rho +2}$, while the kurtosis equals $Ku\left(X\right)=\frac{3{\left(\rho +2\right)}^{2}}{\rho (\rho +4)}$, which is a decreasing function of ρ (yet always taking on higher values than 3), and tends to *∞* as $\rho \to 0^{+}$.

**Figure 3:**
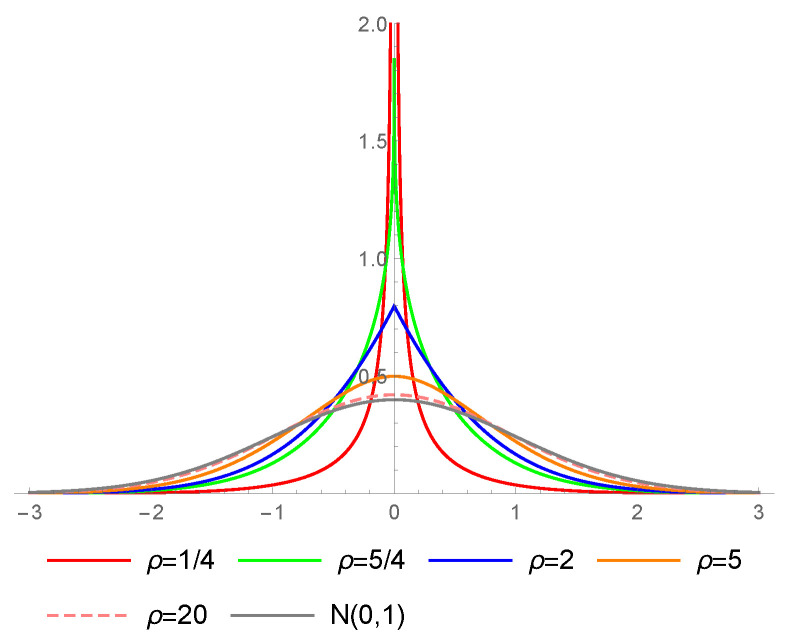
Probability density functions of the MN type I distribution for different values of ρ.

Figure 3 presents the pdf of the MN type I distribution for different values of ρ, compared with the Gaussian curve. Presented cases exhibit the ability of this distribution to capture leptokurtic data. It is straightforward to show that, for ρ<2, the pdf is a convex function on sets (0,∞) and (−∞,0). If 1<ρ<2 (see Figure 3, green line), the density is pointed at zero, thus not differentiable at this point. If ρ≤1, the pdf tends to *∞* as x→0, and degenerates to a single-point distribution as ρ→0. Hence, for ρ≤1, the normalized probability $\epsilon^{-1}P\left(|X|<\epsilon \right)$ tends to *∞* for ϵ→0. For ρ>1, this probability equals $\sqrt{\frac{2}{\pi }}\rho /\left(\rho -1\right)$, approaching *∞* as $\rho \to 1^{+}$.

Note that the accumulation of the mass near to zero is typical for returns of many financial assets, corresponding with only small or even no price changes at all. However, such a behaviour of the data can be observed only locally, in the sense that for a finite sample size it seems a little far-fetched to assume that the normalized probability $\epsilon^{-1}P\left(|X|<\epsilon \right)$ is unbounded. Note that for a finite sample size, such a property for ρ≤1 becomes an assumption (somewhat incidental) that could not be validated. To weaken this assumption and control for the pointedness of the distribution, we introduce a locally leptokurtic distribution with a continuous pdf function at zero, for which $\epsilon^{-1}P\left(|X|<\epsilon \right)$ is bounded on ϵ>0 for any fixed ρ>0, including ρ≤1. Moreover, the pdf for such a distribution is differentiable on the entire real line.

The idea for constructing a locally leptokurtic distribution is to use the censored random variable *R* as a standard deviation of *X*, which (conditionally on *R*) is normally distributed:

(16) $$X|R∼N(0,\max\left\{R^{2},c^{2}\right\}),$$

where *R* is a continuous random variable with Beta(ρ,1) distribution and c∈(0,1) is a censoring parameter. We refer to the marginal distribution of *X* as the local modulated normal (LMN) type I distribution. Its pdf and cdf are given by the following formulas:

(17) $$\begin{array}{cc} pdf(x) & =\int_{0}^{c}\frac{R^{\rho -1}\left(\rho e^{-\frac{x^{2}}{2c^{2}}}\right)}{\sqrt{2\pi }c}\;dR+\int_{c}^{1}\frac{R^{\rho -1}\left(\rho e^{-\frac{x^{2}}{2R^{2}}}\right)}{\sqrt{2\pi }R}\;dR\\ \; & =\left\{\begin{array}{cc} \frac{2^{-\frac{\rho }{2}}\rho {\left(\frac{1}{x^{2}}\right)}^{\frac{1}{2}-\frac{\rho }{2}}\left(\Gamma \left(\frac{1}{2}-\frac{\rho }{2},\frac{x^{2}}{2}\right)-\Gamma \left(\frac{1}{2}-\frac{\rho }{2},\frac{x^{2}}{2c^{2}}\right)\right)+\sqrt{2}c^{\rho -1}e^{-\frac{x^{2}}{2c^{2}}}}{2\sqrt{\pi }}, & x\neq 0\\ \frac{c\rho -c^{\rho }}{\sqrt{2\pi }c\left(\rho -1\right)}, & x=0. \end{array}\right. \end{array}$$

(18) $$cdf\left(y\right)=\left\{\begin{array}{cc} \frac{y\left(cE_{\frac{\rho +1}{2}}\left(\frac{y^{2}}{2}\right)-c^{\rho }E_{\frac{\rho +1}{2}}\left(\frac{y^{2}}{2c^{2}}\right)\right)}{2\sqrt{2\pi }c}-\frac{1}{2}\mathrm{erfc}\left(\frac{y}{\sqrt{2}}\right)+1, & y\neq 0\\ \frac{1}{2}, & y=0. \end{array}\right.$$

Note that the pdf is continuous on the entire real line. Since the first term of the above cdf function tends to 0 as ρ→∞, the LMN type I distribution reduces to standard normal distribution in this limiting case, for any fixed c∈(0,1). Moreover, it reduces to a zero-mean Gaussian case with variance $c^{2}$, when $\rho \to 0^{+}$.

The characteristic function of the LMN type distribution admits the form: $c^{\rho }e^{-\frac{1}{2}c^{2}t^{2}}-\frac{1}{2}\rho \left(E_{1-\frac{\rho }{2}}\left(\frac{t^{2}}{2}\right)-c^{\rho }E_{1-\frac{\rho }{2}}\left(\frac{c^{2}t^{2}}{2}\right)\right)$. The moments $E(X^{2r})$ exist for any ρ>0, c∈(0,1), and are given as $E\left(X^{2r}\right)=\frac{2^{r}\Gamma \left(r+\frac{1}{2}\right)\left(2rc^{\rho +2r}+\rho \right)}{\sqrt{\pi }\left(\rho +2r\right)}$, r∈N. Hence, the variance equals $E\left(X^{2}\right)=\frac{2c^{\rho +2}+\rho }{\rho +2}$, while the kurtosis $Ku\left(X\right)=\frac{3{\left(\rho +2\right)}^{2}\left(4c^{\rho +4}+\rho \right)}{\left(\rho +4\right){\left(2c^{\rho +2}+\rho \right)}^{2}}$, being higher than 3, and approaching this value as either $\rho \to 0^{+}$ or ρ→∞.

**Figure 4:**
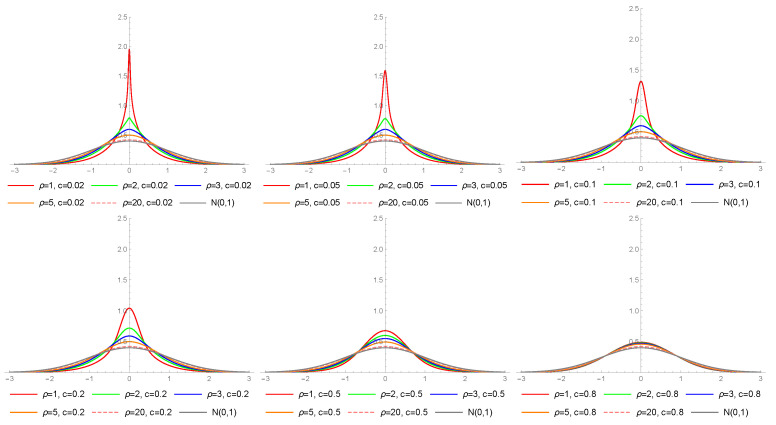
Probability density functions of the LMN type I distribution for different values of ρ≥1, c∈{0.02,0.05,0.1,0.2,0.5,0.8}.

**Figure 5:**
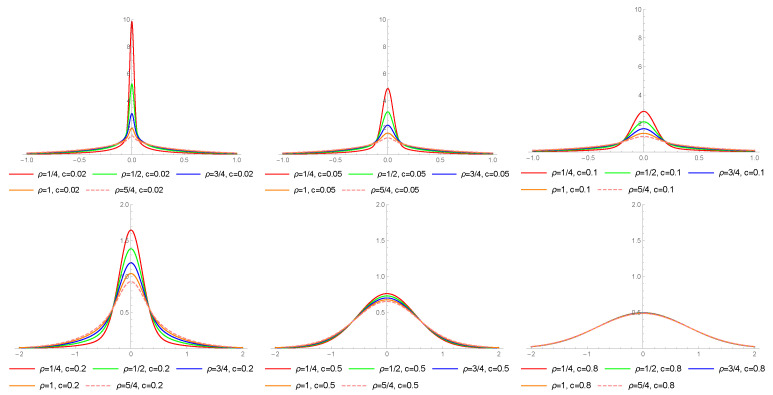
Probability density functions of the LMN type I distribution for different values of ρ≤5/4, c∈{0.02,0.05,0.1,0.2,0.5,0.8}.

Figure 4 presents the densities of the LMN type I distribution for different values of ρ≥1, while Figure 5—for ρ≤5/4, with c∈{0.02,0.05,0.1,0.2,0.5,0.8} in both cases. The presented curves exhibit visible flexibility in capturing leptokurtic data. In particular, it can be noticed that a combination of a small value of ρ with a small value of *c* can produce an “extremely” leptokurtic distribution (see Figure 5, with c=0.02).

### 2.4. Locally Both Leptokurtic and Fat-Tailed Distribution

To capture both effects in financial modelling, that is, leptokurticity and fat tails, we construct a new distribution, belonging to the SMN family, that combines the advantages of both the LS and LMN type I distributions introduced above. Let the conditional distribution of some random variable *X* be defined as:

(19) $$X|R∼N(0,\min\left\{\max\left\{R^{2},c^{2}\right\},d^{2}\right\}),$$

where *R* is a mixture of Beta(ρ,1) and Pareto(1,ν) distributions with weights *p* and 1−p, respectively, i.e.,

(20) $$R=\left\{\begin{array}{cc} Beta(\rho ,1), & \mathrm{with}\;\mathrm{probability}\;p\\ Pareto(1,\nu ), & \mathrm{with}\;\mathrm{probability}\;1-p. \end{array}\right.$$

**Figure 6:**
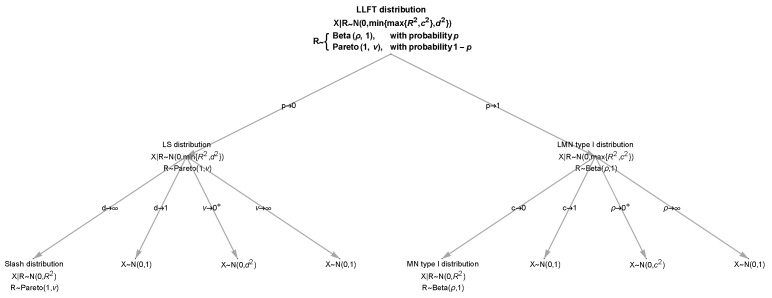
The tree with limiting cases for the LLFT distribution.

We name the marginal distribution of *X* as the locally both leptokurtic and fat-tailed distribution (LLFT, in short), since it has the ability to be (locally) leptokurtic and (locally) heavy-tailed. Note that LLFT distribution can be written as a mixture of the LS distribution (given by (5)) and LMN type I distribution given by (16) with weights *p* and 1−p. The pdf, cdf, characteristic function and moments follow immediately from the previous two sections and well-known properties of probability distributions’ mixtures; therefore, we omit the details. Figure 6 presents the limiting cases of the LLFT distribution, the set of which comprises the slash, LS, MN type I, LMN type I and Gaussian distributions.

**Figure 7:**
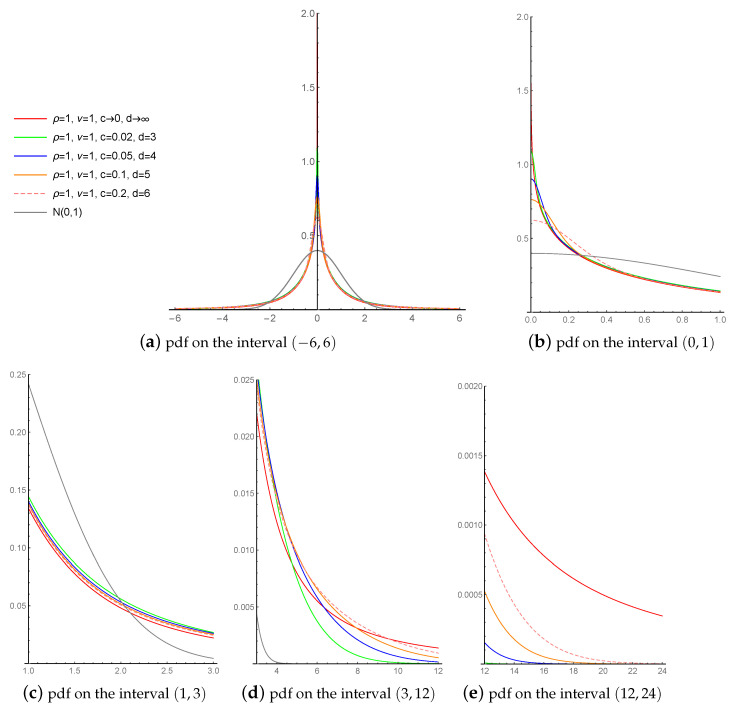
The pdf of the LLFT distribution for different values of parameters *c* and *d* and fixed ν, ρ and p=1/2.

Figure 7 compares the LLFT distribution’s pdf for different values of the censoring parameters $c\in \{0^{+},0.02,0.05,0.1,0.2\}$ and d∈{3,4,5,6,∞}, under fixed ν=1, ρ=1 and p=1/2. Parameter *c* clearly affects the pointedness of the pdf curve: the lower the value of *c*, the more peaked the pdf (see Figure 7b). Moving away from the mode, the curves behave similarly (see Figure 7c), with their tails’ local heaviness driven by the parameter *d*: the higher its values, the longer the tails keep their local heaviness (see Figure 7d), before they thin out (see Figure 7e). This, however, does not pertain to the case when d→∞ (red line), which represents a simple mixture of the slash and MN type I distributions (without their “local” modifications).

As regards the moments of the LLFT distribution, they can easily be derived as the moments of a mixture, and thus we skip their presentation. However, for a reason clarified below, it merits a mention that the expectation $E(e^{rX})$ exists for any r∈N and equals

(21) $$\begin{array}{c} E\left(e^{rX}\right)=1+\sum_{k=1}^{\infty }\left(p\cdot g_{k}+\left(1-p\right)\cdot f_{k}\right)<\infty , \end{array}$$

where

$$g_{k}=\frac{2^{-k}r^{2k}\left(2kc^{2k+\rho }+\rho \right)}{\left(2k+\rho )\Gamma (k+1\right)}$$

and

$$f_{k}=\left\{\begin{array}{cc} \frac{2^{-k}d^{-\nu }r^{2k}\left(2kd^{2k}-\nu d^{\nu }\right)}{\left(2k-\nu )\Gamma (k+1\right)}, & \;\mathrm{for}\;\;\;2k\neq \nu \\ \frac{2^{-k}r^{2k}\left(2k\log\left(d\right)+1\right)}{\Gamma (k+1)}, & \;\mathrm{for}\;\;\;2k=\nu . \end{array}\right.$$

It can be shown that the finiteness of $E(e^{rX})$ stems from the tails of X∼LLFT thinning out beyond the censoring and actually converging to the tails of a zero-mean normal distribution with variance $d^{2}$.

The above feature of the LLFT distribution could prove essential in empirical finances, particularly option pricing, where predicting the price of a derivative hinges upon some models built for logarithmic rates return, say $y_{t}$, the conditional distribution of which (given the past of the process) belongs to the same family of distributions as the one assumed for the standardized errors of the observations. Transformation of the return $y_{t}$ into the price requires using the exponential function for $y_{t}$. Depending on the family distribution of the latter, conditional (on the past) expectation of the price simply may not exist (as it happens in the case of $y_{t}$ following a Student’s *t* or slash distribution). However, from (21), it follows that if the conditional distribution of $y_{t}$ is LLFT, then the expectation of the induced price distribution is finite (conditionally on the past price).

## 3. The SV Model with Locally Leptokurtic and Fat-Tailed Innovations

In this section, we use the newly proposed, LLFT distribution (given by (19) and (20)) in the context of modelling financial time series. In volatility modelling of this type of data, two major classes of models are used: the generalized autoregressive conditionally heteroscedastic (GARCH) and stochastic volatility (or stochastic variance, SV) models. Here, we focus on the latter class. The stochastic variance models were introduced to describe time-varying volatility ([17,24,28,29,30,31]), and it seems that they are more flexible than GARCH. In the basic SV model, a log-normal auto-regressive process is specified for the conditional variance, with the conditional mean equation’s innovations following the Gaussian distribution. We generalize such a model by assuming the LLFT distribution instead, which yields the LLFT-SV specification. Due to the LLFT distribution’s properties (see Section 2), such a model is a natural and promising alternative to the heavy-tailed SV (including t-SV) models proposed in, e.g., [14,17].

The basic stochastic volatility model is defined as follows:

(22) $$y_{t}=x_{t}\beta +\varepsilon_{t}\sqrt{h_{t}},\;$$

(23) $$\ln h_{t}=\gamma +\phi \left(\ln h_{t-1}-\gamma \right)+\eta_{t},$$

where $y_{t}$ denotes an observable at time *t*, $x_{t}$ is an 1×r vector of exogenous variables or lagged observations with parameters comprised in an r×1 vector β, $\left\{\varepsilon_{t}\right\}∼iiN\left(0,1\right)$ is a Gaussian white noise sequence with mean zero and unit variance, $\left\{\eta_{t}\right\}∼iiN\left(0,\;\sigma^{2}\right)$, $\eta_{t}$ and $\varepsilon_{s}$ are mutually independent (denoted as $\;\eta_{t}⊥\varepsilon_{s}$) for all t,s∈{1,2,…,T},*T* is the length of the modelled time series, and finally, $\;0<\left|\varphi \right|<1$.

We extend the above SV specification by waiving the normality $\varepsilon_{t}$ and replacing it with the LLFT distribution given by (19):

(24) $$\varepsilon_{t}=\lambda_{t}\min\left\{\max\left\{\omega_{t},c\right\},d\right\},$$

where $\left\{\lambda_{t}\right\}∼iiN\left(0,\;1\right)$, $\omega_{t}∼Beta\left(\rho ,1\right)$ with probability *p* and $\omega_{t}∼Pareto\left(1,\nu \right)$ with probability 1−p and $\lambda_{s}⊥\omega_{t}$ for s,t∈{1,2,…,T}, with censoring parameters *c* and *d* such that 0<c<1<d.

The conditional distribution of $y_{t}$ in the LLFT-SV model (given the past of $y_{t}$ and the current latent variables $h_{t}$ and $\omega_{t})$ is determined by the distribution of $\lambda_{t}$, so $y_{t}$ follows the normal distribution with mean $\mu_{t}=x_{t}\beta$ and standard deviation $\sigma_{t}=\sqrt{h_{t}}\;\min\left\{\max\left\{\omega_{t},c\right\},d\right\}$. For $\mu_{t}$ we assume an autoregressive structure of order *m* with a constant: $\mu_{t}=\beta_{0}+\beta_{1}y_{t-1}+\beta_{2}y_{t-2}+\ldots +\beta_{m}y_{t-m}$, where the polynomial $1-\sum_{j=1}^{m}\beta_{j}B^{j}$ has roots outside the unit circle.

### 3.1. Bayesian Setup

The Bayesian statistical model amounts to specifying the joint distribution of all observations, latent variables and parameters. The assumptions presented so far determine the conditional distribution of the observations and latent variables, given the parameters, thus necessitating the marginal distribution of the parameters (the prior or a priori distribution) to be formulated. In the prior structure, we assume mutual independence between parameters $\beta ,\phi ,\gamma ,\sigma^{2},\;p,\nu ,\;\rho ,\;c,\;d$ and use standard prior distributions. The vector β, as well as scalar parameter ϕ, are assumed to have a truncated normal distribution. Parameter γ has a normal prior, whereas for $\sigma^{2}$, we set an inverse gamma prior distribution $IG(\alpha_{\sigma },\;\beta_{\sigma })$ with mean $\beta_{\sigma }/\left(\alpha_{\sigma }-1\right)$ under $\alpha_{\sigma }>1$. For ν we assume a gamma distribution $G(\alpha_{\nu },\;\beta_{\nu })$, with mean $\alpha_{\nu }/\beta_{\nu }$. For ρ, we also assume a gamma distribution, while for parameter *p*—a beta distribution. Finally, for parameters *c* and *d*, we assume inverse Nakagami distributions (see [32]) truncated to intervals (0,1) and (1,∞), respectively. A random variable *x* follows an inverse Nakagami distribution with parameters α,β>0, which we write as x∼INK(α,β), if $x^{2}$ is inverse-gamma distributed with mean $\frac{\beta }{\alpha -1}$ (under α>1) and variance $\frac{\beta }{{\left(\alpha -1\right)}^{2}\left(\alpha -2\right)}$ (under α>2), owing to which sampling from INK(α,β) distribution is straightforward and becomes down sampling from the corresponding IG(α,β) distribution and taking the square root of the draw. The probability density function is given as $f_{INK}\left(x|\alpha ,\beta \right)=\frac{2\beta^{\alpha }}{\Gamma (\alpha )}{\left(\frac{1}{x^{2}}\right)}^{\alpha +\frac{1}{2}}e^{-\beta \frac{1}{x^{2}}}$. The inverse Nakagami distribution has been presented by [32], although in a different parameterization. Note that under *c* and *d* following truncated INK distributions, $c^{2}$ and $d^{2}$ are then inverse-gamma distributed (with corresponding truncations). Notice that for c∈(0,1), a beta prior could be considered instead. However, the resulting full conditional posterior would be far less tractable for MCMC sampling. The exact specifications of these distributions are presented later in this section.

Under a sample $y_{1},y_{2},\ldots ,y_{T}$, where *T* is the sample’s length, we introduce the following matrix notation: $y={\left[y_{1},y_{2},\cdots ,y_{T}\right]}^{\prime }$, $h={\left[h_{1},h_{2},\cdots ,h_{T}\right]}^{\prime }$, $\omega ={\left[\omega_{1},\omega_{2},\cdots ,\omega_{T}\right]}^{\prime }$, $y_{\left(0\right)}=\left[h_{0},y_{-m+1},y_{-m+2},\ldots ,y_{0}\right]$ and

$$X=\left[\begin{array}{c} x_{1}\\ \vdots \\ x_{T} \end{array}\right],\;\mathrm{where}\;x_{t}=\left[y_{t-m+1},y_{t-m+2},\ldots ,y_{t}\right],\;\mathrm{for}\;t=1,2,\ldots ,T.$$

As regards the initial conditions $y_{\left(0\right)}$, for $h_{t}$ we fix $h_{0}=1$, while for $y_{t}$ the first *m* pre-sample observations are used. The following symbols are used:

$f_{N}^{n}\left(x|u,V\right)$—the probability density function of the n-variate normal distribution with mean vector u and positive definite covariance matrix V,

$f_{N}\left(x|a,b\right)$—the probability density function of the normal distribution with mean *a* and variance *b*,

$f_{LN}\left(x|a,b\right)$—the probability density function of the log-normal distribution with mean *a* and variance *b*,

$f_{G}\left(x|a,b\right)$—the probability density function of the gamma distribution with mean a/b,

$f_{IG}\left(x|a,b\right)$—the probability density function of the inverse gamma distribution with mean b/(a−1) for a>1,

$f_{Beta}\left(x|a,b\right)$—the probability density function of the beta distribution with parameters *a* and *b*,

$f_{Pareto}\left(x|a,b\right)$—the probability density function of the Pareto distribution with parameters *a* and *b*,

$I_{\left(a,\;b\right)}\left(x\right)$—the indicator function for the interval (a,b).

Now the Bayesian model can be fully written as:

(25) $$\begin{array}{c} p\left(y,h,\omega ,\beta ,\phi ,\gamma ,\sigma^{2},\;p,\nu ,\;\rho ,\;c,\;d|y_{\left(0\right)}\right)=\prod_{t=1}^{T}f_{N}\left(y_{t}|\mu_{t},h_{t}{\left\{\min\left\{\max\left\{\omega_{t},c\right\},d\right\}\right\}}^{2}\right)\times \\ \times \prod_{t=1}^{T}f_{LN}\left(h_{t}|e^{\gamma +\phi \left(lnh_{t-1}-\gamma \right)+\frac{1}{2}\sigma^{2}},\;{(e}^{\sigma^{2}}-1)e^{2\gamma +2\phi \left(lnh_{t-1}-\gamma \right)+\sigma^{2}}\right)\;\times \\ p\left(\omega \right)p\left(\beta \right)p\left(\phi \right)p\left(\sigma^{2}\right)p\left(\gamma \right)p\left(\;\nu \right)p\left(\rho \right)p\left(c\right)p\left(d\right)p\left(p\right), \end{array}$$

where

$$p\left(\omega \right)=\prod_{t=1}^{T}\left[p\;f_{Beta}\left(\omega_{t}|\rho ,\;1\right)+\left(1-p\right)f_{Pareto}\left(\omega_{t}|1,\nu \right)\right],$$

and the prior distributions are specified as follows:

- $p\left(\beta \right)\propto f_{N}^{k+1}\left(\beta |\mu_{\beta },\Sigma_{\beta }\right)I_{\left(0,1\right)}\left({\left|\lambda_{R}\right|}_{max}\right)\propto e^{-\frac{1}{2}{\left(\beta -\mu_{\beta }\right)}^{{}^{\prime }}\Sigma_{\beta }^{-1}\left(\beta -\mu_{\beta }\right)}I_{\left(0,1\right)}\left({\left|\lambda_{R}\right|}_{max}\right)$, $\lambda_{R}$ is the vector of eigenvalues of the companion matrix, related to the AR form (if exists) with characteristic polynomial $\left(1-\sum_{j=1}^{k}\beta_{j}B^{j}\right)$;
- $p\left(\gamma \right)=f_{N}\left(\gamma |\mu_{\gamma },\sigma_{\gamma }^{2}\right)\propto \;e^{-\frac{1}{2\sigma_{\gamma }^{2}}{\left(\gamma -\mu_{\gamma }\right)}^{2}}$;
- $p\left(\phi \right)\propto f_{N}\left(\phi |\mu_{\phi },\sigma_{\phi }^{2}\right)I_{\left(-1,1\right)}\left(\phi \right)\propto e^{-\frac{1}{2\sigma_{\phi }^{2}}{\left(\phi -\mu_{\phi }\right)}^{2}}I_{\left(-1,1\right)}\left(\phi \right)$;
- $p\left(\sigma^{2}\right)=f_{IG}\left(\sigma^{2}|\alpha_{\sigma },\;\beta_{\sigma }\right)=\frac{\alpha_{\sigma }^{\beta_{\sigma }}}{\Gamma \left(\alpha_{\sigma }\right)}{\;\left(\frac{1}{\sigma^{2}}\right)}^{\alpha_{\sigma }+1}e^{-\beta_{\sigma }\frac{1}{\sigma^{2}}}$;
- $p\left(\nu \right)=f_{G}\left(\nu |\alpha_{\nu },\;\beta_{\nu }\right)=\frac{\alpha_{\nu }^{\beta_{\nu }}}{\Gamma \left(\alpha_{\nu }\right)}\nu^{\alpha_{\nu }-1}e^{-\beta_{\nu }\nu }$;
- $p\left(\;\rho \right)=f_{G}\left(\rho |\alpha_{\rho },\;\beta_{\rho }\right)=\frac{\alpha_{\rho }^{\beta_{\rho }}}{\Gamma \left(\alpha_{\rho }\right)}\rho^{\alpha_{\rho }-1}e^{-\beta_{\rho }\rho }$;
- $p\left(c\right)\propto f_{INK}\left(c|\alpha_{c},\beta_{c}\right)I_{\left(0,1\right)}\propto {\left(\frac{1}{c^{2}}\right)}^{\alpha_{c}+\frac{1}{2}}e^{-\beta_{c}\frac{1}{c^{2}}}I_{\left(0,1\right)}\left(c\right)$;
- $p\left(d\right)\propto f_{INK}\left(d|\alpha_{d},\beta_{d}\right)I_{\left(1,+\infty \right)}\propto {\left(\frac{1}{d^{2}}\right)}^{\alpha_{d}+\frac{1}{2}}e^{-\beta_{d}\frac{1}{d^{2}}}I_{\left(1,+\infty \right)}\left(d\right)$;
- $p\left(p\right)=f_{Beta}\left(p|\alpha_{p},\beta_{p}\right)=\frac{\Gamma \left[\alpha_{p}+\beta_{p}\right]p^{\alpha_{p}-1}{\left(1-p\right)}^{\beta_{p}-1}}{\Gamma \left[\alpha_{p}\right]\Gamma \left[\beta_{p}\right]}I_{\left(0,1\right)}\left(p\right)$.

### 3.2. MCMC Method for the Bayesian LLFT-SV Model

The posterior density function, $p(\theta ,\omega ,h|y,y_{\left(0\right)})$, where θ comprises all the model’s parameters, is proportional to (25) and thus, highly dimensional and non-standard. To make an inference about the parameters and latent variables, relevant numerical methods are needed. In our paper, we resort to a common Markov chain Monte Carlo (MCMC) method, namely the Gibbs algorithm, consisting of sequential sampling from the full conditional posteriors derived from (25), which we present below.

#### 3.2.1. The Full Conditional Posterior Distributions of Parameters

The conditional posterior densities of the LLFT-SV model’s parameters are the following (see (25)):

- $p\left(\beta |y,h,\omega ,\gamma ,\phi ,\sigma^{2},\;\nu ,\;\rho ,\;p,\;c,\;d,y_{\left(0\right)}\right)\propto f_{N}^{k+1}\left(\beta |{\tilde{\mu }}_{\beta },\;{\tilde{\Sigma }}_{\beta }\right)I_{\left(0,1\right)}\left({\left|\lambda_{R}\right|}_{max}\right),$ where ${\tilde{\mu }}_{\beta }={\tilde{\Sigma }}_{\beta }\left(\Sigma_{\beta }^{-1}\mu_{\beta }+X^{{}^{\prime }}\Sigma_{y}^{-1}y\right)$, ${\tilde{\Sigma }}_{\beta }={{(X}^{{}^{\prime }}\Sigma_{y}^{-1}X+\Sigma_{\beta }^{-1})}^{-1}$, $\Sigma_{y}=\mathrm{diag}\left(\sigma_{1}^{2},\sigma_{2}^{2},\ldots ,\sigma_{T}^{2}\right)$, and $\sigma_{t}^{2}=h_{t}{\left\{\min\left\{\max\left\{\omega_{t},c\right\},d\right\}\right\}}^{2}$, for t=1,2,…,T;
- $p\left(\gamma |y,h,\omega ,\beta ,\phi ,\sigma^{2},\;\nu ,\;\rho ,\;p,\;c,\;d,\;y_{\left(0\right)}\right)=f_{N}\left(\gamma |{\tilde{\mu }}_{\gamma },{\tilde{\sigma }}_{\gamma }^{2}\right)$, where ${\tilde{\sigma }}_{\gamma }^{2}={\left[\frac{1}{\sigma_{\gamma }^{2}}+\frac{T{\left(\phi -1\right)}^{2}}{\sigma^{2}}\right]}^{-1}$ and ${\tilde{\mu }}_{\gamma }=\left[\frac{\mu_{\gamma }}{\sigma_{\gamma }^{2}}+\frac{\left(1-\phi \right)}{\sigma^{2}}\sum_{t=1}^{T}\left(\ln h_{t}-\phi \ln h_{t-1}\right)\right]{\tilde{\sigma }}_{\gamma }^{2}$;
- $p\left(\phi |y,h,\omega ,\beta ,\gamma ,\sigma^{2},\;\nu ,\;\rho ,\;p,\;c,\;d,\;y_{\left(0\right)}\right)\propto f_{N}\left(\phi |{\tilde{\mu }}_{\phi },{\tilde{\sigma }}_{\phi }^{2}\right)\;I_{\left(-1,\;1\right)}\left(\phi \right),$ where ${\tilde{\mu }}_{\phi }=$
  $\left[\frac{\mu_{\phi }}{\sigma_{\phi }^{2}}+\frac{1}{\sigma^{2}}\sum_{t=1}^{T}\left(\ln h_{t}-\gamma \right)\left(\ln h_{t-1}-\gamma \right)\right]{\tilde{\sigma }}_{\phi }^{2}$ and ${\tilde{\sigma }}_{\phi }^{2}={\left[\frac{1}{\sigma_{\phi }^{2}}+\frac{\sum_{t=1}^{T}{\left(\ln h_{t}-\gamma \right)}^{2}}{\sigma^{2}}\right]}^{-1}$;
- $p\left(\sigma^{2}|y,h,\omega ,\beta ,\gamma ,\phi ,\;\nu ,\;\rho ,p,\;c,\;d,\;y_{\left(0\right)}\right)=f_{IG}\left(\sigma^{2}|{\tilde{\alpha }}_{\sigma },{\tilde{\beta }}_{\sigma }\right),$ where ${\tilde{\alpha }}_{\sigma }=\alpha_{\sigma }+\frac{T}{2}$ and ${\tilde{\beta }}_{\sigma }=\frac{1}{2}\sum_{t=1}^{T}{\left[\ln h_{t}-\gamma -\phi \left(\ln h_{t-1}-\gamma \right)\right]}^{2}+\beta_{\sigma }$;
- $p\left(\rho |y,h,\omega ,\beta ,\gamma ,\phi ,\;\sigma^{2},\nu ,p,\;c,\;d,\;y_{\left(0\right)}\right)=f_{G}\left(\rho |{\tilde{\alpha }}_{\rho },{\tilde{\beta }}_{\rho }\right),$ where ${\tilde{\alpha }}_{\rho }=\alpha_{\rho }+\sum_{t=1}^{T}I_{\left(0,1\right)}\left(\omega_{t}\right)$ and ${\tilde{\beta }}_{\rho }=\beta_{\rho }-\sum_{t=1}^{T}\ln\left[\omega_{t}I_{\left(0,1\right)}\left(\omega_{t}\right)+I_{\left(1,+\infty \right)}\left(\omega_{t}\right)\right]$;
- $p\left(\nu |y,h,\omega ,\beta ,\gamma ,\phi ,\;\sigma^{2},\rho ,\;p,\;c,\;d,\;y_{\left(0\right)}\right)=f_{G}\left(\nu |{\tilde{\alpha }}_{\nu },{\tilde{\beta }}_{\nu }\right),$ where ${\tilde{\alpha }}_{\nu }=\alpha_{\nu }+\sum_{t=1}^{T}I_{\left(1,+\infty \right)}\left(\omega_{t}\right)$ and ${\tilde{\beta }}_{\nu }=\beta_{\nu }+\sum_{t=1}^{T}\ln\left[\omega_{t}I_{\left(1,+\infty \right)}\left(\omega_{t}\right)+I_{\left(0,1\right)}\left(\omega_{t}\right)\right]$;
- $p\left(p|y,h,\omega ,\beta ,\gamma ,\phi ,\;\sigma^{2},\nu ,\;\rho ,\;c,\;d,\;y_{\left(0\right)}\right)=f_{Beta}\left(p|{\tilde{\alpha }}_{p},{\tilde{\beta }}_{p}\right)$, where ${\tilde{\alpha }}_{p}=\alpha_{p}+\sum_{t=1}^{T}I_{\left(0,1\right)}\left(\omega_{t}\right)$ and ${\tilde{\beta }}_{p}=\beta_{p}+\sum_{t=1}^{T}I_{\left(1,+\infty \right)}\left(\omega_{t}\right)$.

Since the above densities of the full conditional distributions have known closed forms, we can directly simulate from these distributions using standard pseudo-random numbers generators.

As regards the parameters *c* and *d*, their full posterior conditionals are far more complicated as compared to the remaining ones.

- Conditional distribution of *c*. If $\sum_{t=1}^{T}I_{\left(0,1\right)}\left(\omega_{t}\right)=0$, then the conditional distribution is straightforward, since $p\left(c|y,h,\omega ,\beta ,\gamma ,\phi ,\sigma^{2},\nu ,\rho ,p,d,y_{\left(0\right)}\right)\propto p\left(c\right)$. If $\sum_{t=1}^{T}I_{\left(0,1\right)}\left(\omega_{t}\right)=n>0$ then the full conditional posterior density of *c* is as follows:
  (26) $$\begin{array}{c} p\left(c|y,h,\omega ,\beta ,\gamma ,\phi ,\sigma^{2},\nu ,\rho ,p,d,y_{\left(0\right)}\right)\propto \\ \frac{1}{\prod_{t=1}^{T}\min\{\max\left\{\omega_{t},c\right\},d\}}\;e^{-\frac{1}{2}\sum_{t=1}^{T}\frac{{\left(y_{t}-x_{t}\beta \right)}^{2}}{h_{t}\min{\left\{\max\left\{\omega_{t},c\right\},d\right\}}^{2}}}\;{\left(\frac{1}{c^{2}}\right)}^{\alpha_{c}+\frac{1}{2}}e^{-\beta_{c}\frac{1}{c^{2}}}I_{\left(0,1\right)}\left(c\right), \end{array}$$
  which can be expressed as a mixture of n+1 different distributions:
  (27) $$\begin{array}{c} p\left(c|y,h,\omega ,\beta ,\gamma ,\phi ,\sigma^{2},\nu ,\rho ,p,d,y_{\left(0\right)}\right)\propto \\ \sum_{k=1}^{n}\underset{g_{k}^{c}\left(c\right)}{\underset{︸}{\frac{1}{\prod_{t=1}^{n}\min\left\{\max\left\{{\tilde{\omega }}_{t},c\right\},d\right\}}\;e^{-\frac{1}{2}\sum_{t=1}^{n}\frac{{\tilde{a}}_{y,t}}{{\min\{\max\left\{{\tilde{\omega }}_{t},c\right\},d\}}^{2}}}\;{\left(\frac{1}{c^{2}}\right)}^{\alpha_{c}+\frac{1}{2}}e^{-\beta_{c}\frac{1}{c^{2}}}I_{\left({\tilde{\omega }}_{k-1},\;{\tilde{\omega }}_{k}\right)}\left(c\right)}}+\\ +\underset{g_{n+1}^{c}\left(c\right)}{\underset{︸}{\frac{1}{c^{n}}e^{-\frac{1}{2}\sum_{t=1}^{n}\frac{{\tilde{a}}_{y,t}}{c^{2}}}\;{\left(\frac{1}{c^{2}}\right)}^{\alpha_{c}+\frac{1}{2}}\;e^{-\beta_{c}\frac{1}{c^{2}}}\;I_{\left({\tilde{\omega }}_{n,},\;1\right)}\left(c\right)}}, \end{array}$$
  where ${\tilde{\omega }}_{1},{\tilde{\omega }}_{2},\ldots ,{\tilde{\omega }}_{T}$ are order statistics, defined by sorting the values of $\omega_{1},\omega_{2},\ldots ,\omega_{T}$ in ascending order, i.e.,
  $$0={\tilde{\omega }}_{0}<{\tilde{\omega }}_{1}<...<\;{\tilde{\omega }}_{n}<1<{\tilde{\omega }}_{n+1}<...<\;{\tilde{\omega }}_{T},$$
  with corresponding ${\tilde{a}}_{y,t}$ for t=1,...,T, where $a_{y,t}=\frac{{\left(y_{t}-x_{t}\beta \right)}^{2}}{h_{t}}$ have been previously assigned to unordered $\omega_{t}$s.
  To simulate from this mixture, the mixing weights and probability density functions with normalizing constants are needed. For k=1, we have a truncated inverse Nakagami distribution for *c* with parameters $\alpha_{c}$ and $\beta_{c}$:
  (28) $$g_{1}^{c}\left(c\right)=w_{c,1}\frac{1}{\prod_{t=1}^{n}{\tilde{\omega }}_{t}}e^{-\left(\frac{1}{2}\sum_{t=1}^{n}\frac{{\tilde{a}}_{y,t}}{{\tilde{\omega }}_{t}^{2}}\right)}\frac{{\left(\frac{1}{c^{2}}\right)}^{\alpha_{c}+\frac{1}{2}}e^{-\beta_{c}\frac{1}{c^{2}}}\;I_{\left(0,\;{\tilde{\omega }}_{1}\right)}\left(c\right)}{w_{c,1}}\;$$
  where $w_{c,1}=\frac{1}{2}\beta_{c}^{-\alpha_{c}}\Gamma \left(\alpha_{c},\frac{\beta_{c}}{{\tilde{\omega }}_{1}^{2}}\right)$ is the normalizing constant for the truncated inverse Nakagami distribution.
  For k=2,...,n, we also have a truncated inverse Nakagami distribution for *c* with parameters $\alpha_{c}+\frac{k-1}{2}$ and $\beta_{c}+\frac{1}{2}\sum_{t=1}^{k-1}{\tilde{a}}_{y,t}$:
  (29) $$g_{k}^{c}\left(c\right)=w_{c,k}\frac{e^{-\frac{1}{2}\sum_{t=k}^{n}\frac{{\tilde{a}}_{y,t}}{{\tilde{\omega }}_{t}^{2}}}}{\prod_{t=k}^{n}{\tilde{\omega }}_{t}}\frac{e^{-\left(\frac{1}{2}\sum_{t=1}^{k-1}{\tilde{a}}_{y,t}+\beta_{c}\right)\frac{1}{c^{2}}}}{w_{c,k}}\;{\left(\frac{1}{c^{2}}\right)}^{\alpha_{c}+\frac{k}{2}}I_{\left(\;{\tilde{\omega }}_{k-1},\;{\tilde{\omega }}_{k}\right)}\left(c\right),$$
  where
  $$\begin{array}{c} w_{c,k}=\frac{1}{2}{\left(\frac{1}{2}\sum_{t=1}^{k-1}{\tilde{a}}_{y,t}+\beta_{c}\right)}^{-\alpha_{c}-\frac{k-1}{2}}\times \\ \times \left\{\Gamma \left(\alpha_{c}+\frac{k-1}{2},\frac{\frac{1}{2}\sum_{t=1}^{k-1}{\tilde{a}}_{y,t}+\beta_{c}}{{\tilde{\omega }}_{k}^{2}}\right)-\Gamma \left(\alpha_{c}+\frac{k-1}{2},\frac{\frac{1}{2}\sum_{t=1}^{k-1}{\tilde{a}}_{y,t}+\beta_{c}}{{\tilde{\omega }}_{k-1}^{2}}\right)\right\}. \end{array}$$
  Finally, for k=n+1:
  (30) $$g_{n+1}^{c}\left(c\right)=w_{c,n+1}\frac{e^{-\left(\frac{1}{2}\sum_{t=1}^{n}{\tilde{a}}_{y,t}+\beta_{c}\right)\frac{1}{c^{2}}}}{w_{c,n+1}}\;{\left(\frac{1}{c^{2}}\right)}^{\alpha_{c}+\frac{n+1}{2}}I_{\left(\;{\tilde{\omega }}_{n},\;1\right)}\left(c\right),$$
  where
  $$\begin{array}{c} w_{c,n+1}=\frac{1}{2}{\left(\frac{1}{2}\sum_{t=1}^{n}{\tilde{a}}_{y,t}+\beta_{c}\right)}^{-\alpha_{c}-\frac{n}{2}}\times \\ \times \left\{\Gamma \left(\alpha_{c}+\frac{n}{2},\frac{\frac{1}{2}\sum_{t=1}^{n}{\tilde{a}}_{y,t}+\beta_{c}}{1}\right)-\Gamma \left(\alpha_{c}+\frac{n}{2},\frac{\frac{1}{2}\sum_{t=1}^{n}{\tilde{a}}_{y,t}+\beta_{c}}{{\tilde{\omega }}_{n}^{2}}\right)\right\} \end{array}$$
  The mixing weights are proportional to: $w_{c,1}^{0}=w_{c,1}\frac{1}{\prod_{t=1}^{n}{\tilde{\omega }}_{t}}e^{-\left(\frac{1}{2}\sum_{t=1}^{n}\frac{{\tilde{a}}_{y,t}}{{\tilde{\omega }}_{t}^{2}}\right)},$
  $w_{c,k}^{0}=w_{c,k}\frac{e^{-\frac{1}{2}\sum_{t=k}^{n}\frac{{\tilde{a}}_{y,t}}{{\tilde{\omega }}_{t}^{2}}}}{\prod_{t=k}^{n}{\tilde{\omega }}_{t}}\;$ for k=2,...,n, and $w_{c,n+1}^{0}=w_{c,n+1}$.
  To simulate from the mixture components, we apply the acceptance-rejection method by using the uniform distribution on the interval $\left({\tilde{\omega }}_{k-1},\;{\tilde{\omega }}_{k}\right)$ for k=1,...,n, and on the interval $\left({\tilde{\omega }}_{n},\;1\right)$ for k=n+1.
- Conditional distribution of *d*. If $\sum_{t=1}^{T}I_{\left(1,+\infty \right)}\left(\omega_{t}\right)=0$, then the conditional distribution is straightforward, since $p\left(d|y,h,\omega ,\beta ,\gamma ,\phi ,\sigma^{2},\nu ,\rho ,p,c,y_{\left(0\right)}\right)\propto p\left(d\right)$. If $\sum_{t=1}^{T}I_{\left(1,+\infty \right)}\left(\omega_{t}\right)=T-n>1$ then the full conditional posterior distribution for *d* can be expressed in a very similar manner:
  (31) $$\begin{array}{c} p\left(d|y,h,\omega ,\beta ,\gamma ,\phi ,\sigma^{2},\nu ,\rho ,p,c,y_{\left(0\right)}\right)\propto \\ \frac{1}{\prod_{t=1}^{T}\min\{\max\left\{\omega_{t},c\right\},d\}}\;e^{-\frac{1}{2}\sum_{t=1}^{T}\frac{{\left(y_{t}-x_{t}\beta \right)}^{2}}{h_{t}\min\{{\max\{\omega_{t},c\},d\}}^{2}}}{\left(\frac{1}{d^{2}}\right)}^{\alpha_{d}+\frac{1}{2}}\;e^{-\beta_{d}\frac{1}{d^{2}}}\;I_{\left(1,+\infty \right)}\left(d\right), \end{array}$$
  which can be expressed as a mixture of T−n+1 different distributions:
  (32) $$\begin{array}{c} p\left(d|y,h,\omega ,\beta ,\gamma ,\phi ,\sigma^{2},\nu ,\rho ,p,c,y_{\left(0\right)}\right)\propto \\ \frac{1}{d^{T-n}}e^{-\frac{1}{2}\sum_{t=n+1}^{T}\frac{{\tilde{a}}_{y,t}}{d^{2}}}{\left(\frac{1}{d^{2}}\right)}^{\alpha_{d}+\frac{1}{2}}e^{-\beta_{d}\frac{1}{d^{2}}}I_{\left(1,\;{\tilde{\omega }}_{n+1}\right)}\left(d\right)+\\ +\sum_{k=2}^{T-n}\frac{1}{\prod_{t=1+n}^{T}\min\left\{\max\left\{{\tilde{\omega }}_{t},c\right\},d\right\}}e^{-\frac{1}{2}\sum_{t=n+1}^{T}{\frac{{\tilde{a}}_{y,t}}{\min\{\max\left\{{\tilde{\omega }}_{t},c\right\},d\}}}^{2}}\times \\ \times {\left(\frac{1}{d^{2}}\right)}^{\alpha_{d}+\frac{1}{2}}e^{-\beta_{d}\frac{1}{d^{2}}}\;I_{\left({\tilde{\omega }}_{n+k-1},\;{\tilde{\omega }}_{n+k}\right)}\left(d\right)+\\ +\frac{1}{\prod_{t=1+n}^{T}{\tilde{\omega }}_{t}}\;e^{-\frac{1}{2}\sum_{t=n+1}^{T}\frac{{\tilde{a}}_{y,t}}{{\tilde{\omega }}_{t}^{2}}}\;{\left(\frac{1}{d^{2}}\right)}^{\alpha_{d}+\frac{1}{2}}e^{-\beta_{d}\frac{1}{d^{2}}}I_{\left({\tilde{\omega }}_{T},\;+\infty \right)}\left(d\right), \end{array}$$
  In this case, the first component is proportional to the truncated inverse Nakagami distribution for *d* with parameters $\alpha_{d}+\frac{T-n}{2}$ and $\beta_{d}+\frac{1}{2}\sum_{t=n+1}^{T}{\tilde{a}}_{y,t}$:
  (33) $$g_{1}^{d}\left(d\right)=w_{d,1}\frac{{\left(\frac{1}{d^{2}}\right)}^{\alpha_{d}+\frac{T-n+1}{2}}\;e^{-\left(\frac{1}{2}\sum_{t=n+1}^{T}{\tilde{a}}_{y,t}+\beta_{d}\right)\frac{1}{d^{2}}}I_{\left(1,\;{\tilde{\omega }}_{n+1}\right)}\left(d\right)}{w_{d,1}}\;$$
  where
  $$\begin{array}{c} w_{d,1}=\frac{1}{2}{\left(\frac{1}{2}\sum_{t=n+1}^{T}{\tilde{a}}_{y,t}+\beta_{d}\right)}^{-\alpha_{d}-\frac{T-n}{2}}\times \\ \times \left\{\Gamma \left(\alpha_{d}+\frac{T-n}{2},\frac{\frac{1}{2}\sum_{t=n+1}^{T}{\tilde{a}}_{y,t}+\beta_{d}}{{\tilde{\omega }}_{n+1}^{2}}\right)-\Gamma \left(\alpha_{d}+\frac{T-n}{2},\frac{\frac{1}{2}\sum_{t=n+1}^{T}{\tilde{a}}_{y,t}+\beta_{d}}{1}\right)\right\} \end{array}$$
  is the normalizing constant for the truncated inverse Nakagami distribution.
  For k=2,...,T−n, we also have a truncated inverse Nakagami distribution for *d* with parameters $\alpha_{d}+\frac{T-n-k+1}{2}$ and $\beta_{d}+\frac{1}{2}\sum_{t=n+k}^{T}{\tilde{a}}_{y,t}$:
  (34) $$\begin{array}{c} g_{k}^{d}\left(d\right)=w_{d,k}\frac{1}{\prod_{t=1+n}^{n+k-1}{\tilde{\omega }}_{t}}\;e^{-\frac{1}{2}\sum_{t=n+1}^{n+k-1}\frac{{\tilde{a}}_{y,t}}{{\tilde{\omega }}_{t}^{2}}}\times \\ \times \frac{{\left(\frac{1}{d^{2}}\right)}^{\alpha_{d}+\frac{1+T-n-k+1}{2}}e^{-\left(\frac{1}{2}\sum_{t=n+k}^{T}{\tilde{a}}_{y,t}+\beta_{d}\right)\frac{1}{d^{2}}}I_{\left({\tilde{\omega }}_{n+k-1,},\;{\tilde{\omega }}_{n+k}\right)}\left(d\right)}{w_{d,k}}, \end{array}$$
  where
  $$\begin{array}{c} w_{d,k}=\frac{1}{2}{\left(\frac{1}{2}\sum_{t=n+k}^{T}{\tilde{a}}_{y,t}+\beta_{d}\right)}^{-\alpha_{d}-\frac{T-n-k+1}{2}}\times \\ \times \left\{\Gamma \left(\alpha_{d}+\frac{T-n-k+1}{2},\frac{\frac{1}{2}\sum_{t=n+k}^{T}{\tilde{a}}_{y,t}+\beta_{d}}{{\tilde{\omega }}_{n+k}^{2}}\right)\right.\\ -\left.\Gamma \left(\alpha_{d}+\frac{T-n-k+1}{2},\frac{\frac{1}{2}\sum_{t=n+k}^{T}{\tilde{a}}_{y,t}+\beta_{d}}{{\tilde{\omega }}_{n+k-1}^{2}}\right)\right\}. \end{array}$$
  The last component is:
  (35) $$g_{T-n+1}^{d}\left(d\right)=\frac{w_{d,k}}{\prod_{t=1+n}^{T}{\tilde{\omega }}_{t}}\;e^{-\frac{1}{2}\sum_{t=n+1}^{T}\frac{{\tilde{a}}_{y,t}}{{\tilde{\omega }}_{t}^{,2}}}\;\frac{{\left(\frac{1}{d^{2}}\right)}^{\alpha_{d}+\frac{1}{2}}e^{-\beta_{d}\frac{1}{d^{2}}}I_{\left({\tilde{\omega }}_{T,},\;+\infty \right)}\left(d\right)}{w_{d,T-n+1}},$$
  where $w_{d,T-n+1}=\frac{1}{2}{\left(\beta_{d}\right)}^{-\alpha_{d}}\;\left\{\Gamma \left(\alpha_{d}\right)-\Gamma \left(\alpha_{d},\frac{\beta_{d}}{{\tilde{\omega }}_{T}^{2}}\right)\right\}.$ In this case, the mixing weights are proportional to: $w_{d,1}^{0}=w_{d,1}$, $w_{d,k}^{0}=\frac{w_{d,k}}{\prod_{t=1+n}^{n+k-1}{\tilde{\omega }}_{t}}\;e^{-\frac{1}{2}\sum_{t=n+1}^{n+k-1}\frac{{\tilde{a}}_{y,t}}{{\tilde{\omega }}_{t}^{2}}}$ for k=2,...,T−n, and $w_{d,T-n+1}^{0}=\frac{w_{d,T-n+1}}{\prod_{t=1+n}^{T}{\tilde{\omega }}_{t}}\;e^{-\frac{1}{2}\sum_{t=n+1}^{T}\frac{{\tilde{a}}_{y,t}}{{\tilde{\omega }}_{t}^{2}}}$.
  Similarly to the algorithm for the parameter *c*, to simulate from the mixture components here, the acceptance–rejection method is applied by using the uniform distribution on the interval $\left({\tilde{\omega }}_{n+k-1},\;{\tilde{\omega }}_{n+k}\right)$ for k=2,...,T−n. In turn, for k=T−n+1, we simulate from a truncated gamma distribution on the interval $\left({\tilde{\omega }}_{T},\;+\infty \right)\;$using the algorithm proposed by [33].
  If $\sum_{t=1}^{T}I_{\left(1,+\infty \right)}\left(\omega_{t}\right)=T-n=1$, then we have only two components in the mixture (32), the first and last ones.

#### 3.2.2. The Conditional Posterior of $h_{t}$s

We sample each element of the vector h using the independence Metropolis–Hastings (similarly to [24]) algorithm within a separate Gibbs step, initializing at $h_{t}=0.1y_{t}^{2}+1$ for t=1,…,T. The conditional posterior density of $h_{t}$, t∈{1,⋯,T}, is given by

(36) $$\begin{array}{c} p\left(h_{t}|y,h_{1},\cdots ,h_{t-1},h_{t+1},\cdots ,h_{T},\omega ,\beta ,\gamma ,\phi ,\sigma^{2},\nu ,\rho ,p,c,d,y_{\left(0\right)}\right)\propto \\ h_{t}^{-1}e^{-\frac{1}{2\sigma_{h}^{2}}{\left(\ln h_{t}-s_{h}\right)}^{2}}\;h_{t}^{-\frac{1}{2}}e^{-\frac{1}{2}\frac{{\left(y_{t}-x_{t}\beta \right)}^{2}}{h_{t}{\left\{\min\left\{\max\left\{\omega_{t},c\right\},d\right\}\right\}}^{2}}}, \end{array}$$

where ${\tilde{\sigma }}_{h}^{2}=\frac{\sigma^{2}}{1+\phi^{2}}$, ${\tilde{s}}_{h}=\gamma +\frac{\phi \left(\ln h_{t-1}\;-\gamma \right)+\phi \left(\ln h_{t+1}\;-\gamma \right)}{1+\phi^{2}}$ for t=1,...,T−1, and ${\tilde{\sigma }}_{h}^{2}=\sigma^{2}$, ${\tilde{s}}_{h}=\gamma +\phi \left(\ln h_{t-1}-\gamma \right)$ for t=T.

The proposal distribution used to draw $h_{t}$ is the inverted gamma:

$$IG\left(\varphi +\frac{1}{2},\left(\varphi -1\right)e^{{\tilde{s}}_{h}+\frac{1}{2}{\tilde{\sigma }}_{h}^{2}}+\frac{1}{2}\frac{{\left(y_{t}-x_{t}\beta \right)}^{2}}{{\left\{\min\left\{\max\left\{\omega_{t},c\right\},d\right\}\right\}}^{2}}\right),$$

where $\varphi =\frac{2e^{{\tilde{\sigma }}_{h}^{2}}-1}{e^{{\tilde{\sigma }}_{h}^{2}}-1}$. As regards the initial conditions for $\ln h_{t}$, it is assumed that $\ln h_{0}=0$.

#### 3.2.3. The Conditional Posterior of $\omega_{t}$s

The conditional posterior density of $\omega_{t}$, t∈{1,⋯,T}, is given by:

(37) $$\begin{array}{c} p\left(\omega_{t}|y,h,\;\omega_{1},\cdots ,\omega_{t-1},\omega_{t+1},\cdots ,\omega_{T},\beta ,\gamma ,\phi ,\sigma^{2},\nu ,\rho ,p,c,d,y_{\left(0\right)}\right)\propto \\ \frac{1}{\min\{\max\left\{\omega_{t},c\right\},d\}}e^{-\frac{1}{2}\frac{{\left(y_{t}-x_{t}\beta \right)}^{2}}{h_{t}{\left\{\min\left\{\max\left\{\omega_{t},c\right\},d\right\}\right\}}^{2}}}\times \\ \times \left[p\omega_{t}^{\rho -1}\rho I_{\left(0,1\right)}\left(\omega_{t}\right)+\left(1-p\right)\nu \omega_{t}^{-(\nu +1)}I_{\left(1,+\infty \right)}\left(\omega_{t}\right)\right], \end{array}$$

which can be written as a mixture of four different distributions:

(38) $$\begin{array}{c} p\left(\omega_{t}|y,h,\omega_{1},\cdots ,\omega_{t-1},\omega_{t+1},\cdots ,\omega_{T},\beta ,\gamma ,\phi ,\sigma^{2},\nu ,\rho ,p,c,d,y_{\left(0\right)}\right)\propto \\ w_{\omega ,1}\;\underset{f_{1}\left(\omega_{t}\right)}{\underset{︸}{{c^{-\rho }\omega }_{t}^{\rho -1}\rho I_{\left(0,c\right)}\left(\omega_{t}\right)}}+p\;\rho \;w_{\omega ,2}\underset{f_{2}\left(\omega_{t}\right)}{\underset{︸}{\frac{\omega_{t}^{\rho -2}e^{-\frac{1}{2}a_{y,t}}}{w_{\omega ,2}}I_{\left(c,1\right)}\left(\omega_{t}\right)}}+\\ +\left(1-p\right)\;\nu \;w_{\omega ,3}\underset{f_{3}\left(\omega_{t}\right)}{\underset{︸}{\frac{{\left(\frac{1}{\omega_{t}^{2}}\right)}^{\frac{\nu }{2}+1+\frac{1}{2}}e^{-}\frac{1}{2}a_{y,t}\frac{1}{\omega_{t}^{2}}}{w_{\omega ,3}}I_{\left(1,d\right)}\left(\omega_{t}\right)}}+\\ +w_{\omega ,4}\underset{f_{4}\left(\omega_{t}\right)}{\underset{︸}{d^{\nu }\;\nu \;{\left(\frac{1}{\omega_{t}}\right)}^{\nu +1}I_{\left(d,+\infty \right)}\left(\omega_{t}\right)}}\propto \sum_{i=1}^{4}{\tilde{w}}_{\omega ,i}f_{i}\left(\omega_{t}\right), \end{array}$$

where $w_{\omega ,1}=\;c^{\rho -1}\;p\;e^{-\frac{1}{2}\frac{{\left(y_{t}-x_{t}\beta \right)}^{2}}{h_{t}c^{2}}},\;a_{y,t}=\frac{{\left(y_{t}-x_{t}\beta \right)}^{2}}{h_{t}},$

$w_{\omega ,2}=a_{y,t}^{\frac{\rho }{2}-\frac{1}{2}}\;2^{-\frac{\rho }{2}-\frac{1}{2}}\;\left[\Gamma \left(\frac{1}{2}-\frac{\rho }{2},\frac{a_{y,t}}{2}\right)-\Gamma \left(\frac{1}{2}-\frac{\rho }{2},\frac{a_{y,t}}{2c^{2}}\right)\right],$

$w_{\omega ,3}=a_{y,t}^{-\frac{\nu }{2}-\frac{1}{2}}\;2^{\frac{\nu }{2}-\frac{1}{2}}\;\left[\Gamma \left(\frac{\nu }{2}+\frac{1}{2},\frac{a_{y,t}}{2d^{2}}\right)-\Gamma \left(\frac{\nu }{2}+\frac{1}{2},\frac{a_{y,t}}{2}\right)\right],$

$w_{\omega ,4}=\left(1-p\right)\;d^{-\nu -1}\;e^{-\frac{1}{2}\frac{{\left(y_{t}-x_{t}\beta \right)}^{2}}{h_{t}d^{2}}}.$

The weights ${\tilde{w}}_{\omega ,i}$, i=1,2,3,4, of the mixture are as follows:

${\tilde{w}}_{\omega ,i}=\frac{w_{\omega ,i}}{w_{\omega ,1}+p\rho w_{\omega ,2}+\left(1-p\right)\nu w_{\omega ,3}+w_{\omega ,4}}$ for i=1,4,

${\tilde{w}}_{\omega ,2}=\frac{p\rho w_{\omega ,2}}{w_{\omega ,1}+p\rho w_{\omega ,2}+\left(1-p\right)\nu w_{\omega ,3}+w_{\omega ,4}}$,

and ${\tilde{w}}_{\omega ,3}=\frac{\left(1-p\right)\nu w_{\omega ,3}}{w_{\omega ,1}+p\rho w_{\omega ,2}+\left(1-p\right)\nu w_{\omega ,3}+w_{\omega ,4}}$.

It is easy to build an algorithm to generate from the mixture. First, we sample from the uniform distribution to randomly select the component distribution, and then we generate a pseudo-random value from it. The first term of the mixture is given by the density:

$$f_{1}\left(\omega_{t}\right)=c^{-\rho }\omega_{t}^{\rho -1}\rho I_{\left(0,c\right)}\left(\omega_{t}\right),$$

which can be sampled using the inverse cdf technique. The second density is:

$$f_{2}\left(\omega_{t}\right)=\frac{\omega_{t}^{\rho -2}e^{-\frac{1}{2}a_{y,t}}}{w_{\omega ,2}}I_{\left(c,1\right)}\left(\omega_{t}\right),$$

which for ρ>1 is the truncated beta density with parameters ρ−1 and 1. However, for 0<ρ<1, we obtain a non-standard distribution, but an independence Metropolis–Hastings algorithm can be applied with the proposal distribution being the Beta distribution with parameters ρ+1 and 1. The third term of the mixture is:

$$f_{3}\left(\omega_{t}\right)=\frac{{\left(\frac{1}{\omega_{t}^{2}}\right)}^{\frac{\nu }{2}+1+\frac{1}{2}}e^{-}\frac{1}{2}a_{y,t}\frac{1}{\omega_{t}^{2}}}{w_{\omega ,3}}I_{\left(1,d\right)}\left(\omega_{t}\right),$$

which is the density of an inverse gamma distribution of $\omega_{t}^{2}$, truncated to the interval (1,d). Thus, we can sample $\omega_{t}^{2}$ from the truncated inverted gamma distribution with shape parameters $\frac{\nu }{2}+1$ and scale parameters $\frac{1}{2}a_{y,t}$, and then calculate $\omega_{t}$. We simulate from the truncated gamma distribution using the algorithm proposed by [33]. The last term of the mixture is given by the following probability density function:

$$f_{4}\left(\omega_{t}\right)=d^{\nu }{\left(\frac{1}{\omega_{t}}\right)}^{\nu +1}\rho I_{\left(d,+\infty \right)}\left(\omega_{t}\right),$$

which can be easily sampled from via the inverse cdf technique. We initialize the sampler at starting values $\omega_{t}=1.1$ for t=1,…,T.

## 4. Empirical Illustrations

In this section, we apply the LLFT-SV model to describe the volatility of three time series of daily stock market prices. We begin with the analysis of a quite particular asset, namely the MS Industries AG (MSAG.DE), which is a Germany-based industrial technology company. The MSAG.DE data set is specific in the sense of featuring many (repeating) zero returns, which is typical for many individual companies’ stocks. Section 4.1 and Section 4.2 cover the results for the original and perturbed prices series, respectively, with a very detailed analysis providing in-depth insights into the LLFT-SV model performance. Then, in Section 4.3, we shift our attention to two common stock market indices: S&P 500 and DAX, to examine the LLFT-SV model’s validity also for more ‘typical’ financial data.

The MSAG.DE stock prices were downloaded from http://finance.yahoo.com (accessed on 31 March 2021) and cover the period from 28 December 2016 through 25 February 2021. The series is transformed into logarithmic rates returns, expressed in percentage points, and forming a series of 1053 observations. The first two available observations are spared for the initial condition in the AR(2) structure underlying the mean equation (two lags are chosen in view of the Lindley type test for the restriction that the autocorrelation parameters for higher lags are equal to zero). Therefore, $x_{t}=\left(1,y_{t-1},y_{t-2}\right)$, and the final number of the modelled observation is T=1051.

**Figure 8:**
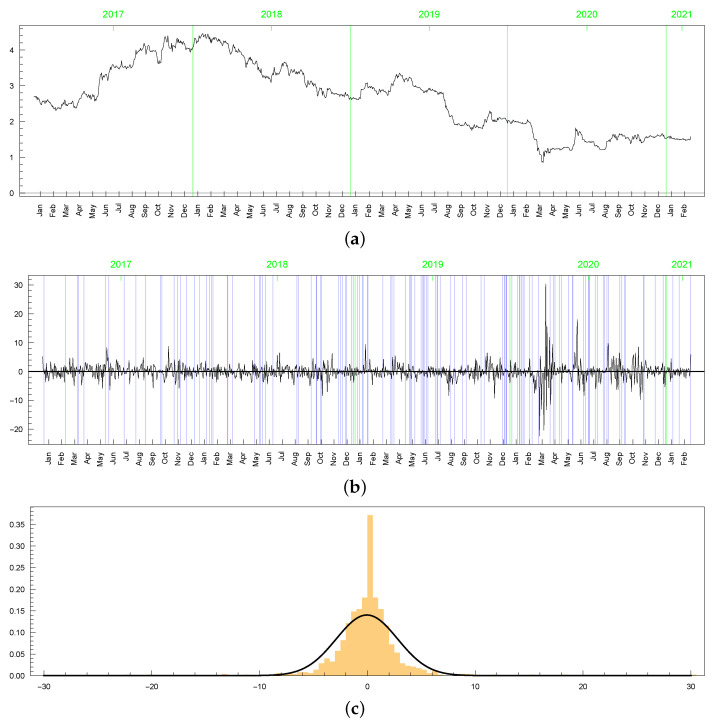
Time plots of (**a**) daily prices, (**b**) log returns in percentages for MSAG.DE, (**c**) histogram of log returns (in percentages) for MSAG.DE with a Gaussian curve.

Basic descriptive characteristics of the modelled data are presented in Table 1, with the prices and returns plotted in Figure 8. It can be seen from the graphs that the return rates are centred around zero, featuring some outliers, as well. The data distribution is highly non-Normal, as confirmed by kurtosis, which exceeds three by far. From Table 1, it can also be noted that the returns’ range is fairly spread, with their minimum and maximum at −22.399 and 30.390, respectively. These extreme observations occurred in March 2020 and can be explained by the turbulence in financial markets caused by the COVID-19 pandemic. The data are also characterized by the presence of zero returns, with zero being exactly the median (while the sample mean is close to zero). The relative frequency of zero returns is equal to about 0.11. The exact positions of the zero returns are indicated in the figures by the blue vertical lines. This relatively high concentration of the zero returns manifests in the histogram through a prominent peak at the mode (see panel (c) in Figure 8). It is worth noting that the zero returns are not always related to zero volumes. In many cases, the volume is positive, but the price stays unchanged.

In view of the above, it could be argued that the data at hand, with some concentration in zero, should be modelled by means of mixed, continuously-discrete distributions. These, however, remain highly unpopular in practice, usually giving way to models based on families of continuous distributions. As pointed out by [27], Bayesian inference on the basis of a sample containing repeated observations (not necessarily zero-valued) is not always possible with the use of a continuous sampling distribution, even under a proper prior distribution. Admittedly, the LLFT-SV model introduced in this paper, as being based on a mixture of continuous probability distributions, fails to explicitly model the incidence of zero returns. However, owing to the properties of the LLFT distribution proposed in this paper, in the LLFT-SV model, the zero returns or any repeated observations do not pose such a problem, since (under the assumed prior structure) it can be proved that the marginal likelihood (i.e., marginal data density value) is finished (see Theorem A1 in Appendix A). The empirical study is divided into two subsections. In the first one, we analyze the original series, featuring repeated zero returns. However, the validity of the LLFT-SV model can also be illustrated in the case where repeated observations do no occur per se, but the series still contains values that concentrate tightly around the median. To this end, we perturb the MSAG.DE prices and present the results for such a series in Section 4.2.

For the sake of comparison, we also consider the Stochastic Volatility model with the conditional Student’s *t*-distribution (t-SV). The *t*-distribution’s density function can be expressed as a scale mixture of normals. That is, the *t*-distributed (with ν degrees of freedom) random variable $\varepsilon_{t}$ in the t-SV model can be written as follows: $\varepsilon_{t}=\lambda_{t}\sqrt{\omega_{t}}$, where $\left\{\lambda_{t}\right\}∼iiN\left(0,\;1\right)$ and $\left\{\omega_{t}\right\}∼iiIG\left(\frac{\nu }{2},\frac{\nu }{2}\right)$. The t-SV model is usually used to better explain the heavy tails of the empirical distributions of returns. However, as exemplified below, in contrast to LLFT-SV, the t-SV model may still not be flexible enough to simultaneously allow for fat tails and a very high peak close to the mean (or median) returns.

We set the following values of the hyperparameters of the model under consideration: $\mu_{\beta }=0$, $\Sigma_{\beta }=I$, $\mu_{\gamma }=0$, $\sigma_{\gamma }=10$, $\mu_{\phi }=0$, $\sigma_{\phi }=10$, $\alpha_{\sigma }=2.5$, $\beta_{\sigma }=0.16$ (see [34]), $\alpha_{\rho }=10$, $\beta_{\rho }=10$, $\alpha_{p}=1$, $\beta_{p}=1$.

Such prior distributions reflect our rather little prior knowledge about the model’s parameters. In order to examine the sensitivity of the posterior distributions to the priors, we try different values of the remaining hyperparameters, which we present in subsections below.

To obtain a pseudo-random sample from the posterior distribution, we use the hybrid sampler presented in the previous section—the Metropolis–Hastings algorithm within the Gibbs procedure, generating 50,000 of burn-in and 200,000 posterior drawings. The algorithm has been implemented in the authors’ own computer code written in GAUSS. Computations were carried out on a personal computer with an Intel Core i7-9850H processor and 16 GB RAM and took about 5–6 h for a single model. A detailed examination of the MCMC convergence is provided in Appendix B.

### 4.1. Results for the Original MSAG.DE Data

In this subsection, we present the results obtained for the original MSAG.DE data set and assumea priori that the values of *c* very close to 0 are unlikely. To represent such a prior belief about *c*, it is assumed that c∼INK(2;0.1) truncated to the interval (0,1).

Table 2 reports the posterior medians, 90% confidence intervals, and interquartile ranges. The 90% confidence intervals are calculated using the 5th and 95th percentiles of the posterior samples.

**Figure 9:**
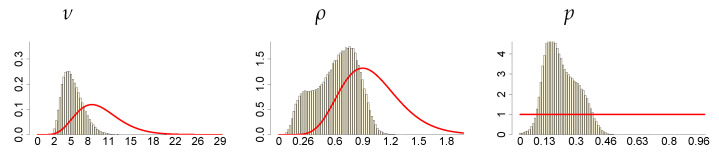
Histograms of the marginal posteriors (bars) and priors (red line) of mixture parameters ν, ρ and *p*, under ν∼G(8;0.8), c∼INK(2;0.1) and d∼INK(2;100).

**Figure 10:**
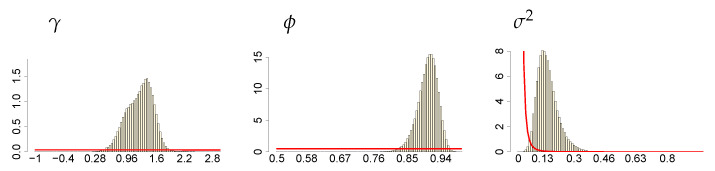
Histograms of the marginal posteriors (bars) and priors (red line) of SV parameters γ, ϕ and $\sigma^{2}$, under ν∼G(8;0.8), c∼INK(2;0.1) and d∼INK(2;100).

**Figure 11:**
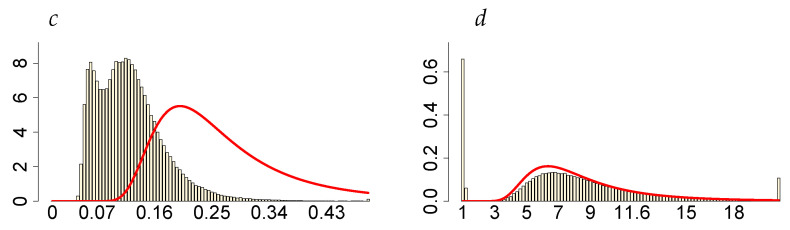
Histograms of the marginal posteriors (bars) and priors (red line) of *c* and *d*, under ν∼G(8;0.8), c∼INK(2;0.1) and d∼INK(2;100).

**Figure 12:**
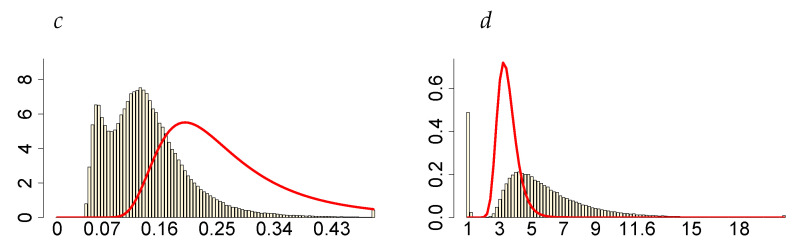
Histograms of the marginal posteriors (bars) and priors (red line) of *c* and *d*, under ν∼G(0.2;0.05), c∼INK(2;0.1) and d∼INK(9;100).

**Figure 13:**
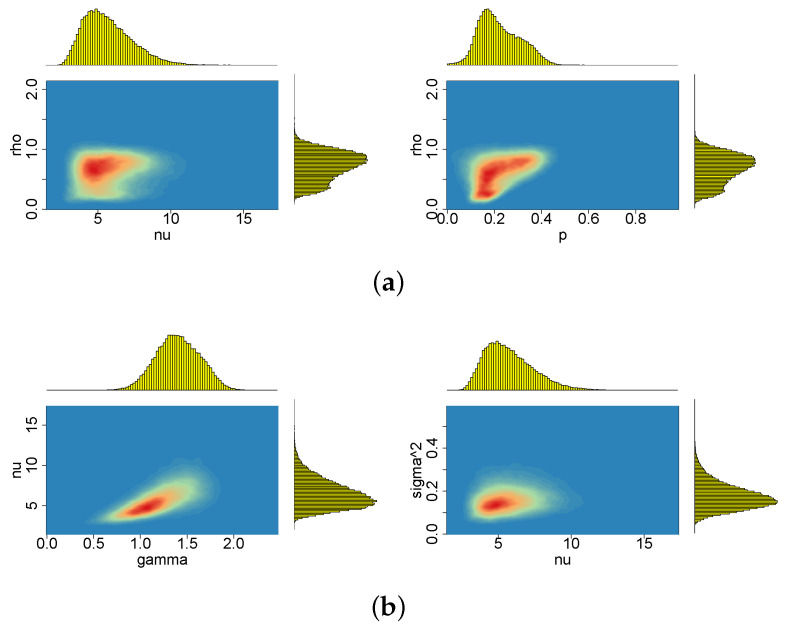
Bivariate marginal posteriors: (**a**) for pairs (ν,ρ) and (p,ρ), (**b**) for pairs (γ,ν) and $\left(\nu ,\sigma^{2}\right)$; obtained in the LLFT-SV model with ν∼G(8;0.8), c∼INK(2;0.1) and d∼INK(2;100).

**Figure 14:**
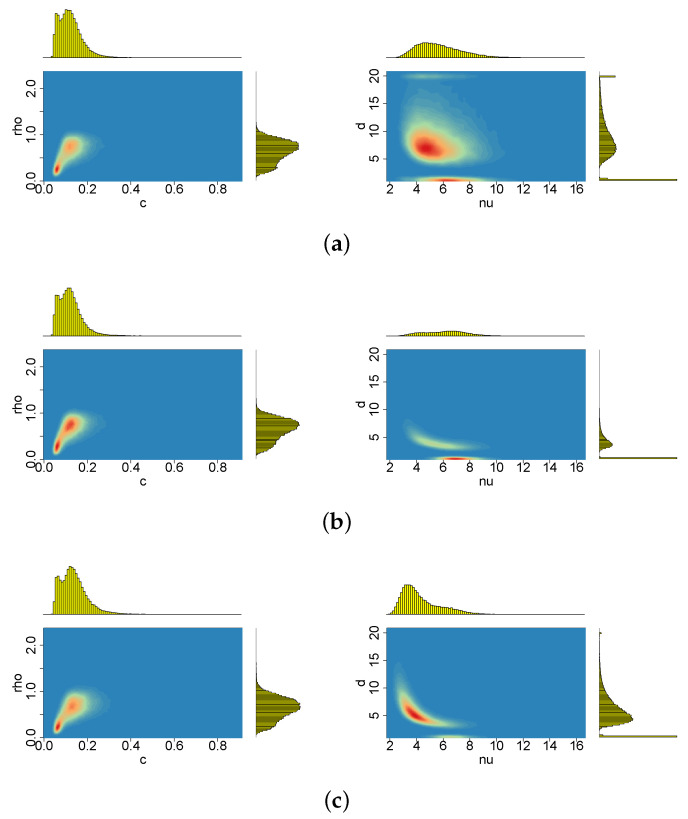
Bivariate marginal posteriors for pairs (c,ρ) and (ν,d), obtained in the LLFT-SV model with (**a**) ν∼G(8;0.8), c∼INK(2,0.1), d∼INK(2;100), (**b**) ν∼G(8;0.8), c∼INK(2,0.1), d∼INK(9;100), (**c**) ν∼G(0.2;0.05), c∼INK(2,0.1), d∼INK(9;100).

Figure 9, Figure 10, Figure 11, Figure 12, Figure 13 and Figure 14 depict the posterior densities of the model parameters obtained under several different prior distributions. Figure 9, Figure 10, Figure 11 and Figure 12 show univariate marginal posteriors and priors. Clear differences between the priors and posteriors indicate that the data contribute significant information about the parameters, changing our prior assumptions. The dependencies between ν and *d*, as well as between ρ and *p*, visible in Figure 13 and Figure 14, are striking. The plots reveal nonlinear relations and multimodality. Moreover, the posterior distributions of *d* and ν are very sensitive to the prior distributions (compare the first three columns by pairs from Table 2). The sensitivity of the posterior distribution of ν with respect to the prior can also be seen in the t-SV model (see the last two columns in Table 2). However, in both types of models considered here, the percentiles of ν indicate that the local slash and Student’s *t*-distributions are more appropriate than the normal distribution for the mean equation innovations. In turn, the posterior distribution of parameter *p* indicates that the locally leptokurtic and heavy-tailed mixture normal distribution is more appropriate than the local slash distribution. The LLFT distribution makes it possible to explain both the outlying returns and the returns concentrating close to their median. As regards the persistence in volatility (ϕ) and the variance of the volatility process ($\sigma^{2}$), all models give similar, though not identical, results.

**Figure 15:**
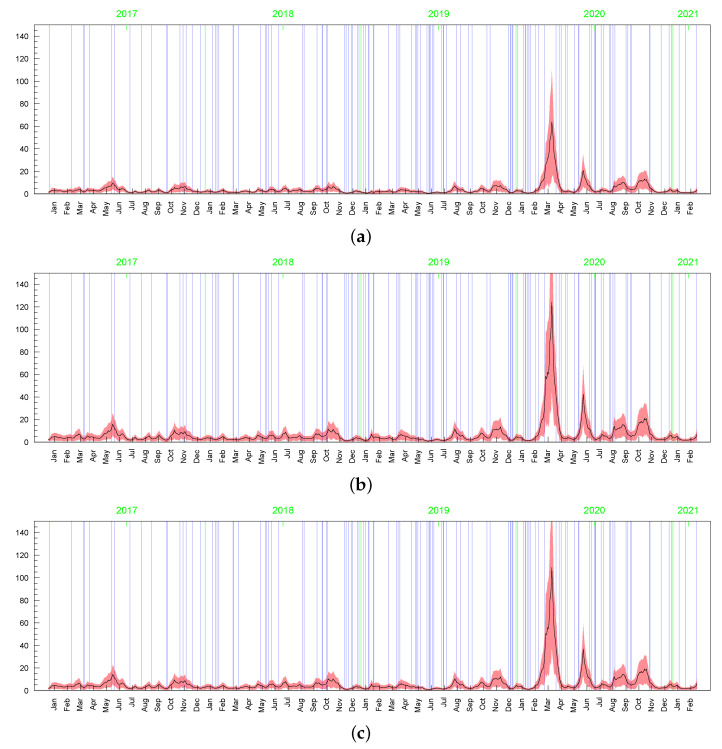
Posterior means (black line) with two standard-deviation bands (truncated only to positive values; red) of $h_{t}$s obtained in the LLFT-SV model with: (**a**) ν∼G(0.2;0.05), c∼INK(2,0.1), d∼INK(9;100), (**b**) ν∼G(8;0.8), c∼INK(2,0.1), d∼INK(9;100), (**c**) ν∼G(8;0.8), c∼INK(2,0.1), d∼INK(2;100).

**Figure 16:**
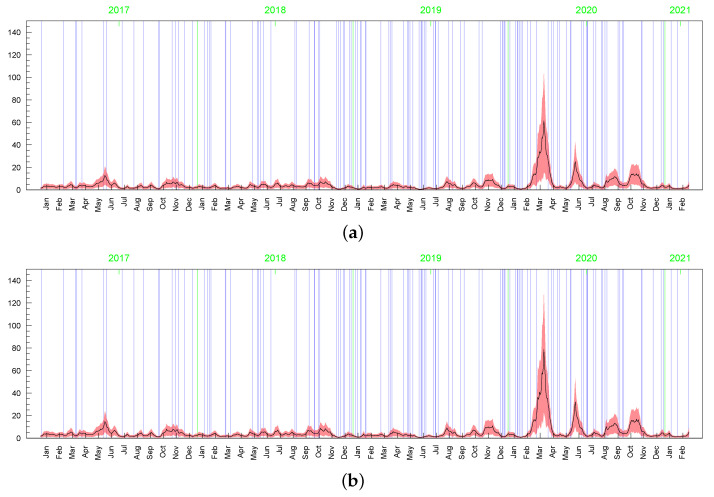
Posterior means (black line) with two standard-deviation bands (truncated only to positive values; red) of $h_{t}$s obtained in the t-SV model with: (**a**) ν∼G(0.2;0.05), (**b**) ν∼G(8;0.8).

**Figure 17:**
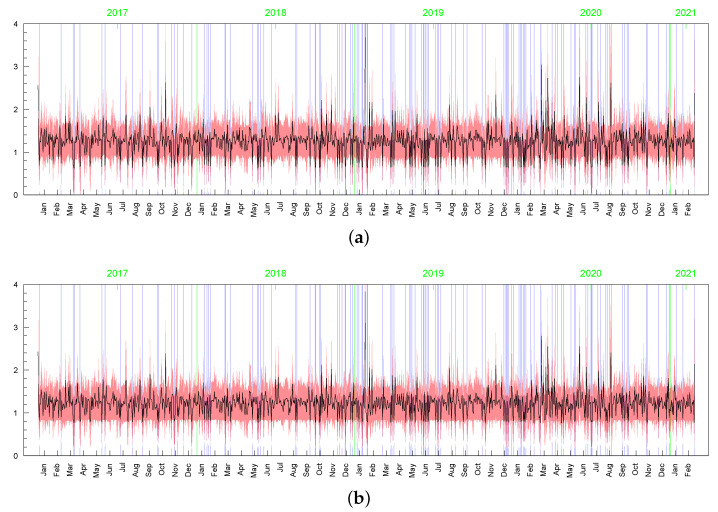
Posterior means (black line) with two-standard-deviation bands (truncated only to positive values; red) obtained in the LLFT-SV model with ν∼G(0.2;0.05), c∼INK(2;0.1), d∼INK(9;100) for: (**a**) $\omega_{t}$, (**b**) $\min\{\max\left\{\omega_{t},c\right\},d\}$.

**Figure 18:**
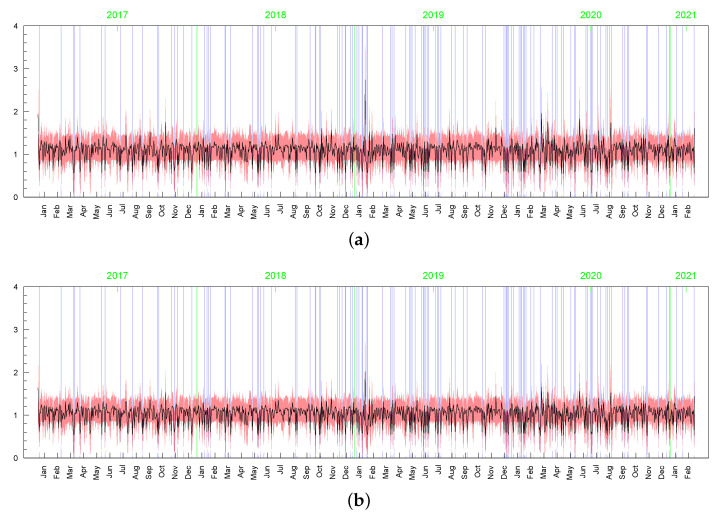
Posterior means (black lines) with two-stsandard-deviation bands (truncated only to positive values; red) of $\min\{\max\left\{\omega_{t},c\right\},d\}$, obtained in the LLFT-SV model with ν∼G(8;0.8), c∼INK(2,0.1), and: (**a**) d∼INK(2;100), (**b**) d∼INK(9;100).

**Figure 19:**
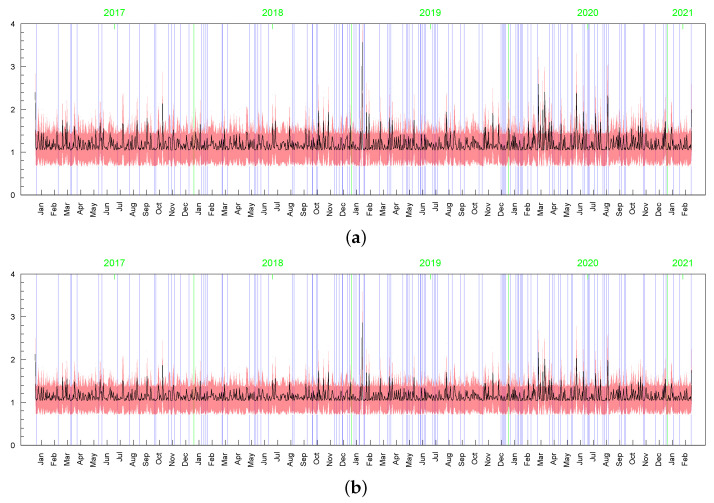
Posterior means (black line) with two-standard-deviation bands (truncated only to positive values; red) of $\sqrt{\omega_{t}}$ obtained in the t-SV model with: (**a**) ν∼G(0.2,0.05), (**b**) ν∼G(8,0.8).

**Figure 20:**
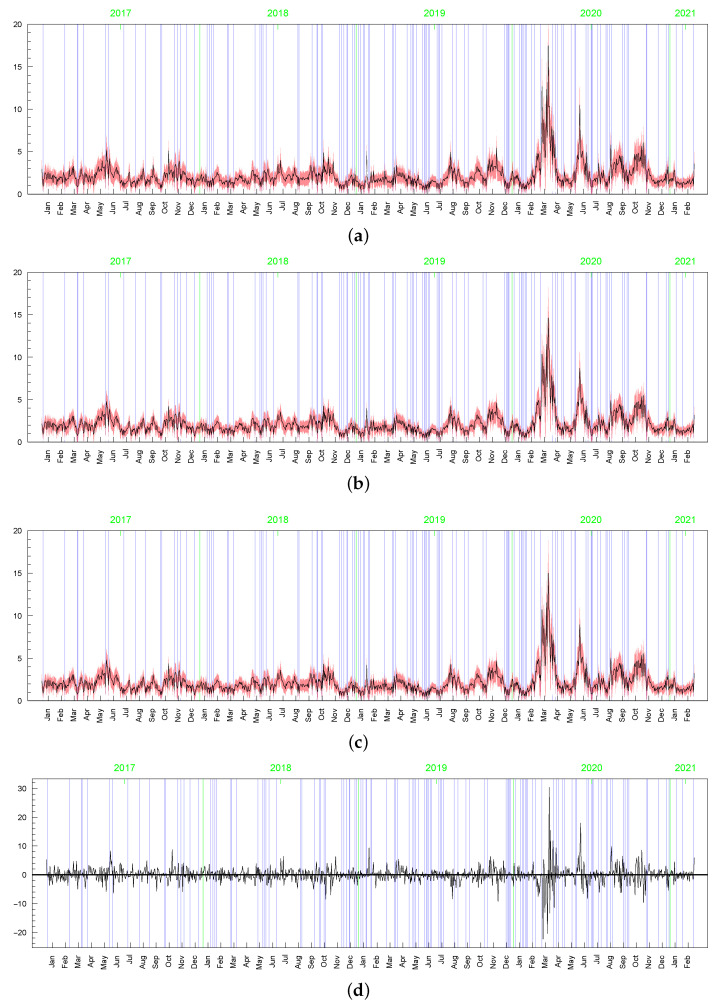
Posterior means (black line) with two-standard-deviation bands (truncated only to positive values; red) of $\sigma_{t}$ obtained in the LLFT-SV model with: (**a**) ν∼G(0.2,0.05), c∼INK(2,0.1), d∼INK(9;100), (**b**) ν∼G(8,0.8), c∼INK(2,0.1), d∼INK(9;100), (**c**) ν∼G(8,0.8), c∼INK(2,0.1), d∼INK(2;100). (**d**) The series of modelled returns.

**Figure 21:**
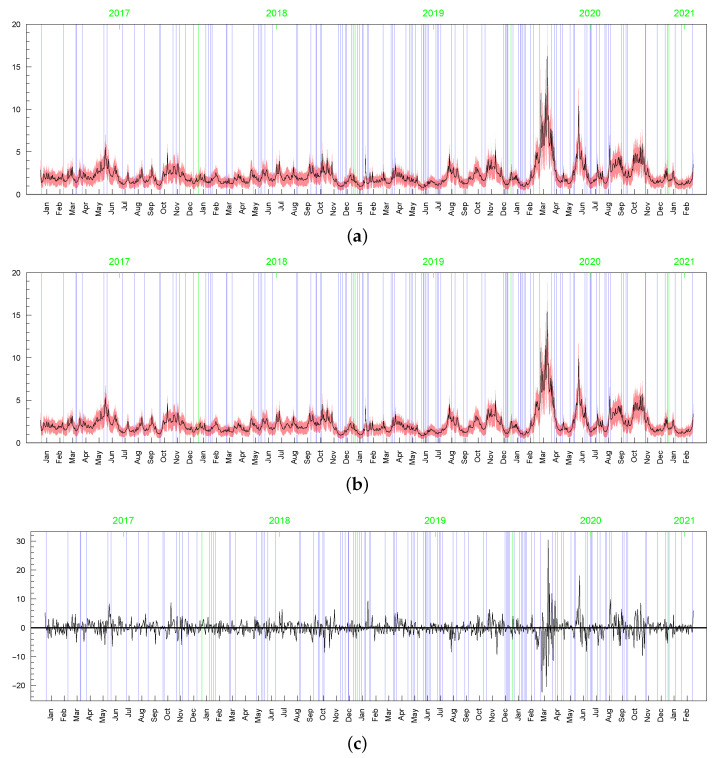
Posterior means (black line) with two-standard-deviation bands (truncated only to positive values; red) of $\sigma_{t}$ obtained in the t-SV model with: (**a**) ν∼G(0.2;0.05), (**b**) ν∼G(8;0.8). (**c**) The series of modelled returns.

However visible, the posterior sensitivity of the latent processes’ parameters does not transfer into similar sensitivity of the posterior means of the processes themselves, especially as it comes to the conditional standard deviations of the returns, $\sigma_{t}$ (see Table 3). The average of posterior means of $\sigma_{t}=\sqrt{h_{t}}\min\left\{\max\left\{\omega_{t},c\right\},d\right\}$ is equal to 2.119 with the standard deviation 1.051. In the t-SV model, the average of posterior means of $\sigma_{t}=\sqrt{h_{t}\omega_{t}}$ is equal to 2.173 with the standard deviation 1.210. The series of the posterior means of $\sigma_{t}$ obtained in both the models are highly correlated, with the correlation coefficient at 0.964. In all Bayesian models considered in this subsection, the corresponding latent processes are highly correlated, indicating their very similar dynamics. It can be seen in Figure 15, Figure 16, Figure 17, Figure 18, Figure 19, Figure 20 and Figure 21, where we graphed the posterior means and conditional standard deviations of $h_{t}$s (in both of the models $h_{t}$s are relatable) and $\sqrt{\omega_{t}}$ in t-SV that corresponds to $\min\{\max\left\{\omega_{t},c\right\},d\}$ in LLFT-SV. Notice that due to the non-negativity of these quantities, the two-standard-deviation bands presented in the figures are truncated only to positive values.

Finally, we compare the predictive capabilities of the Bayesian LLFT-SV and t-SV models. For this purpose, the data set is split into two subsets: training and ex post prediction evaluation sets. As the training set, we take the first 800 observations, with the resulting posterior that can actually be viewed as the prior distribution before a consecutive observation is included into the recursive (not rolling) estimation window. The forecast evaluation period encompasses the most recent 251 trading days. We perform one- to ten-step-ahead predictions over the period 2 March 2020 through 25 February 2021, which gives 242 predictive distributions for each of the ten forecast horizons under consideration, and thereby 2420 predictive distributions in total. The predictive distributions are calculated based on the whole data set available at time 800+t for each t=1,⋯,242 (recursive forecasting scheme). The models are re-estimated each time, i.e., upon arrival of each new observation. Each of the predictive densities is based upon 50,000 MCMC posterior draws, preceded by either 250,000 burn-in passes for t=1 or 10,000 cycles for t=2,3,…,242, with the sampler each time initiated at the final draw of the previous run. The sequence of the one-step-ahead predictive distributions covers the period from 2 March 2020 to 12 February 2021, while the sequence of the ten-step-ahead predictive distributions covers the period 2 March 2020–25 February 2021.

As the basis of the predictive model comparison, we choose the sum of the so-called log predictive likelihoods, which for some horizon *h* in a model *M* can be written as:

(39) $$SLP\left(h,M\right)=\sum_{t=\tilde{T}+1}^{\tilde{T}+n_{h}}\log p(y_{t+h-1}^{o}|y_{1}^{t-1,o},y_{\left(0\right)},M),$$

where $p(y_{t+h}^{o}|y_{1}^{t,o},y_{\left(0\right)},M)$ is the predictive density of $y_{t+h}$ at the observed value $y_{t+h}^{o}$, $y_{1}^{t,o}={\left(y_{1}^{o},...,y_{t}^{o}\right)}^{\prime }$ denotes the observations up to time *t*, $n_{h}$ is the number of forecasts, and finally, $\tilde{T}=T-n_{h}$. Note that for h=1, the difference: $SLP\left(1,M_{1}\right)-SLP\left(1,M_{2}\right)$ amounts to the cumulative log predictive Bayes factor in favour of model $M_{1}$ against $M_{2}$, which informs us how the posterior chances of $M_{1}$ versus $M_{2}$ (based on the observations up to time $\tilde{T}$) change upon observing predicted data, $y_{\tilde{T}+1}^{\tilde{T}+n_{h}}={\left(y_{\tilde{T}+1},...,y_{\tilde{T}+n_{h}}\right)}^{\prime }$.

The use of predictive likelihoods (the predictive density values at the realized “future” observations) are motivated and described in, e.g., [35]. Note that, in this study, we do not consider the models’ in-sample “fit”, which is not only due to our intent to avoid the burden of efficient approximation of marginal data density values (and the issue of sensitivity with respect to the priors) but mostly because it would be hardly related to the present context of forecasting.

The predictive density values (predictive likelihoods) in Equation (39) need to be calculated numerically using draws from the posterior distribution of parameters and latent variables:

$$\begin{array}{c} \hat{p}\left(y_{t+h-1}^{o}|y_{1}^{t-1,o},y_{\left(0\right)},M\right)=\frac{1}{N}\sum_{j=1}^{N}p\left(y_{t+h-1}^{o}|y_{1}^{t-1,o},\theta^{\left(j\right)},h_{1}^{t+h-1,(j)},\omega_{1}^{t+h-1,(j)},y_{\left(0\right)},M\right), \end{array}$$

where $\theta^{\left(j\right)}$, $h_{1}^{t+h-1,(j)}={\left(h_{1}^{\left(j\right)},\cdots ,h_{t+h-1}^{\left(j\right)}\right)}^{\prime }$, $\omega_{1}^{t+h-1,(j)}={\left(\omega_{1}^{\left(j\right)},\cdots ,\omega_{t+h-1}^{\left(j\right)}\right)}^{\prime }$ are draws from the posterior (j=1,2,…,N).

Table 4 presents the results of predictive power comparisons of two alternative models: LLFT-SV and t-SV, obtained for the original (i.e., not perturbed) data set and assuming that ν∼G(8;0.8) and fixed values of parameters *c* and *d* are the medians of their marginal posteriors, obtained under two different priors for *c* (we provide further details below). Thus, two cases are further considered. In the first one: c=0.118 and d=7.553, while in the second: c=0.014 and d=2.808. Note that we decided to fix the values of these two parameters here (rather than sample them from their posterior), which is intended to spare much of otherwise largely time-consuming calculations. Even now, the entire forecasting exercise for a single LLFT-SV model, with fixed values of *c* and *d*, takes as much as roughly two days of calculations (on a personal computer with Intel Core i7-9850H processor and 16 GB RAM). Otherwise, allowing for posterior sampling of these two parameters as well would extend this time to about eight days (for a single model).

The results in Table 4 show clear evidence that the LLFT-SV models (particularly the one with c=0.014) outperform t-SV for all forecast horizons. Due to apparently small differences in the log predictive likelihoods of the t-SV and LLFT-SV models with c=0.118 and d=7.553, one could get an impression that both are quite comparable when it comes to their predictive power. However, the cumulative decimal logarithm of the predictive Bayes factor in favour of the LLFT-SV model with c=0.118 versus t-SV, defined as a difference of the corresponding sums of the log predictive likelihoods for h=1 (see the column for h=1 in Table 4), is equal to about 2.175, which indicates that the posterior odds ratio based on the first 800 observations and calculated in favour of the LLFT-SV model increased by about 150 times upon observing the predicted data. The LLFT-SV model with c=0.014 provides further, even more considerable improvement, since the cumulative logarithm of the predictive Bayes factor in favour of this model against the one with c=0.118 equals about 22. Thus, the posterior odds of these two models increases by as much as about 22 orders of magnitude upon observing data over the forecast period (i.e., 2 March 2020 through 25 February 2021). Note that the low value of c=0.014 allows $\sigma_{t}$ to reach very small values (close to zero), thus enabling the distribution of the conditional mean equation’s innovations to feature a noticeable peakedness. Therefore, it may be concluded that enabling a sharp peak at the mode in the conditional distribution and modelling returns hovering near zero seem to be more important to the predictive performance than fat tails (at least for the data at hand).

### 4.2. Results for the Perturbed MSAG.DE Data

Now, we illustrate the validity of the LLFT-SV model in the case where repeated observations do no occur per se, but the series still contains values that concentrate tightly around (but not at) the mode. For this purpose, we use slightly perturbed observations, using two kinds of perturbation applied to the price series, both in the spirit of [27]. In the first case, the data are perturbed by adding a uniformly distributed random number from the interval $\left(-5\times 10^{-5},5\times 10^{-5}\right)$, while we widen the interval to $\left(-5\times 10^{-4},5\times 10^{-4}\right)$ in the second. As a consequence of the price perturbation, the perturbed returns become different from each other and different from zero. The perturbed returns corresponding to the zero returns in the original series fall within the intervals (−0.0072,0.0049) and (−0.056,0.058) in the first and second case, respectively. Basic descriptive characteristics (not presented in the paper for the sake of brevity) of the perturbed daily returns are very similar to those of the original data, presented in Table 1. Moreover, the histograms of the original and both the perturbed data sets practically coincide (thus, we do not present them, either). Naturally, the medians are now different from zero, equaling −0.0005 in the first case and 0.0034 in the second. In the first case, the concentration of the returns around zero is higher than in the second case, with about 11% of the returns lying within the interval (−0.0072,0.0049), and only 3.2% in the second case (in the same interval).

It can be seen from Table 5 that upon specifying INK(2;0.1) as the prior for *c* in the LLFT-SV model, thus preferring a priori values of the parameter to be relatively distant from zero, the results of Bayesian inference about parameters and latent variables turns out to be quite robust to the considered perturbations. It seems that under such a prior, the values of *c* are too high, dominating (i.e., censoring) $\omega_{t}$, to “adequately” explain the presence of numerous observations very close to the mode. Thereby, the returns that are close but not equal to zero appear to still be treated here in the same way as the zero returns in the model for the original series.

Turning back to Table 5, we notice that very similar results are obtained for the t-SV model. It seems that although the model “adequately” captures the heavy tails of the empirical distribution of the returns, it fails to explain the observations that are compressed around the mode. In both types of models, the inference about latent processes, $h_{t}$ and $\sigma_{t}$, remains unchanged (again, relevant graphs are not presented in the paper, for the sake of brevity).

Since both fat tails and a high concentration of financial returns around the mode need to be accounted for simultaneously, we intend to also consider such LLFT-SV models that allow *c* to be closer to zero (as compared with the prior assumptions in the previous analysis). Recall that smaller values of *c* lead to a more pointed distribution of the conditional mean equation’s innovations. To investigate this fact, we assume that *c* follows a priori the NIK(0.4;0.009) distribution, so that its mode is now equal to about 0.1 instead of 0.2 considered in the previous analysis. Simultaneously, we impose the restriction ν≥1, which is necessary from a numerical point of view. This restriction is not needed when *c* is bounded away from zero (then the posterior probability of ν≤1 is equal to zero).

**Figure 22:**
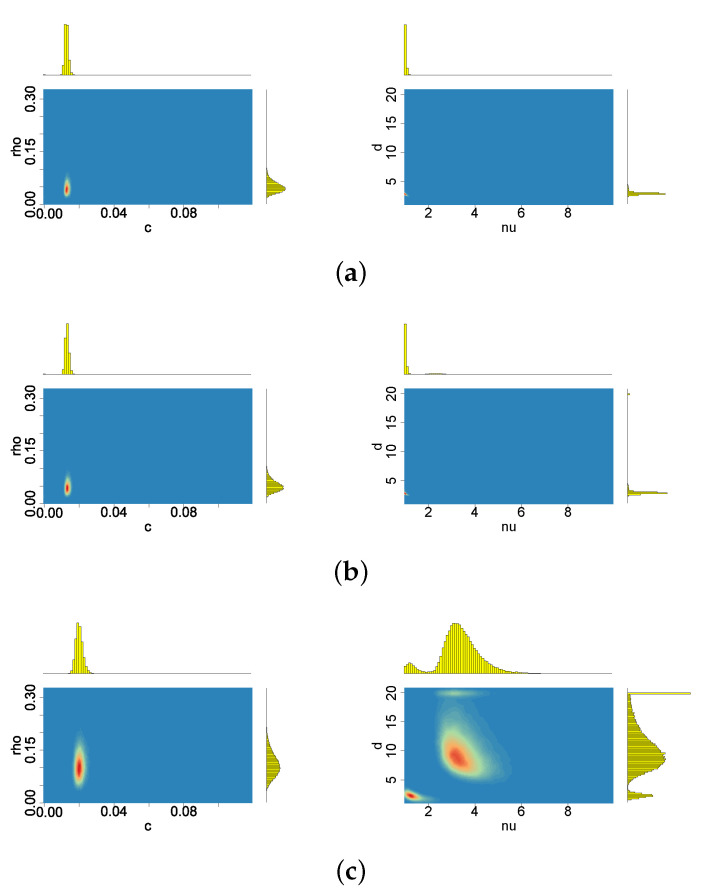
Bivariate marginal posteriors for pairs (c,ρ) and (ν,d), obtained in the LLFT-SV model, under ν∼G(8;0.8), c∼INK(0.4;0.009) and d∼INK(2;100); (**a**) for the original MSAG.DE data set, (**b**) for the perturbed data, $U(-5\times 10^{-5},5\times 10^{-5})$, (**c**) for the perturbed data, $U(-5\times 10^{-4},5\times 10^{-4})$.

When the prior distribution of *c* admits relatively more mass close to zero, only the larger perturbation (the second one) turns out to change the location and dispersion of the posterior distributions ν and ρ, which also affects, in consequence, the inference about all the remaining parameters (apparently with the exception of the volatility persistence, ϕ), see Table 6. For the original data and the first perturbation, the two parameters: ν and ρ, have their posterior probability mass at much lower values than in the case of c∼INK(2;0.1). Moreover, the posterior marginals of these two parameters exhibit a very high concentration around their modes. Additionally, *d* gathers almost all of its posterior mass near 1 (see Figure 22), indicating that the presence of the latent process $h_{t}$ in the LLFT-SV structure is enough to describe heavy tails of the empirical distribution of modelled returns.

**Figure 23:**
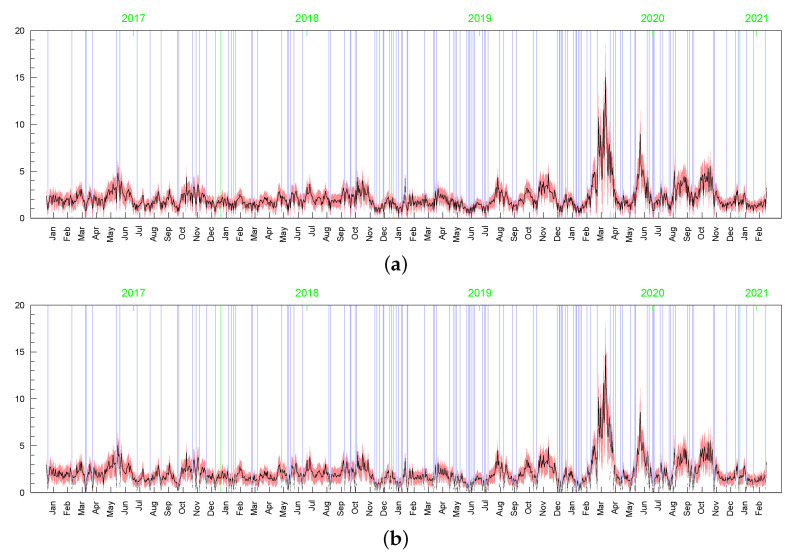
Posterior means (black line) with two-standard-deviation bands (truncated only to positive values; red) of $\sigma_{t}$ obtained for the original MSAG.DE data in the LLFT-SV model with ν∼G(8;0.8), and: (**a**) c∼INK(2;0.1), (**b**) c∼INK(0.4;0.009).

**Figure 24:**
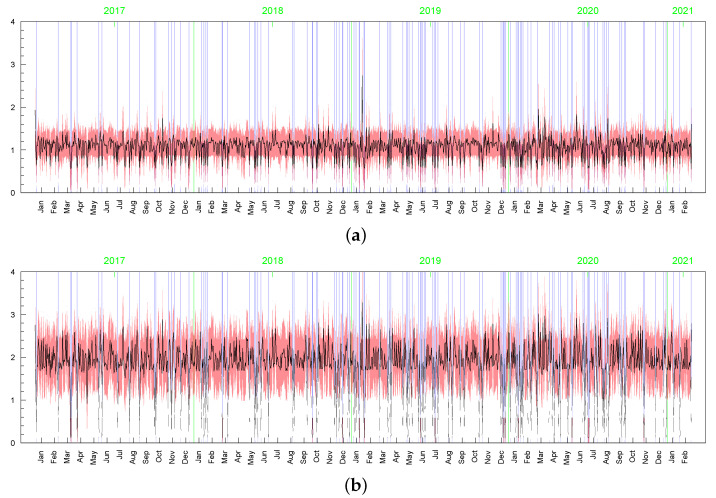
Posterior means (black line) with two-standard-deviation bands (truncated only to positive values; red) of $\min\{\max\left\{\omega_{t},c\right\},d\}$, obtained for the original MSAG.DE data in the LLFT-SV model with ν∼G(8;0.8), and: (**a**) c∼INK(2;0.1), (**b**) c∼INK(0.4;0.009).

**Figure 25:**
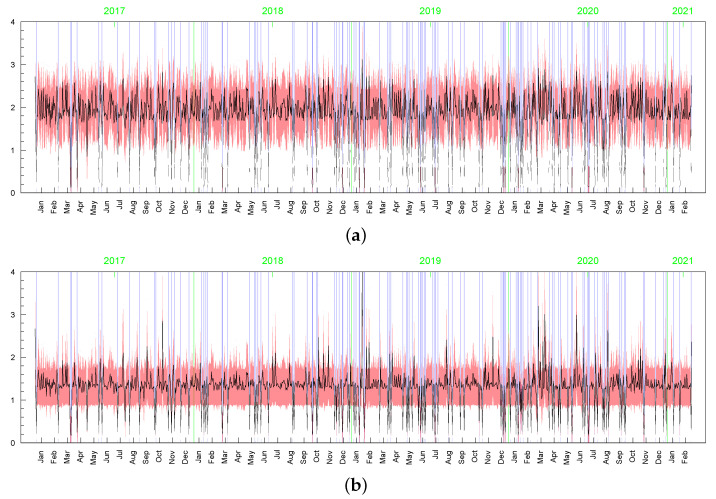
Posterior means (black line) with two-standard-deviation bands (truncated only to positive values; red) of $\min\{\max\left\{\omega_{t},c\right\},d\}$, obtained in the LLFT-SV model for the perturbed MSAG.DE data. The prior distribution: ν∼G(8;0.8), c∼INK(0.4;0.009). The result for: (**a**) the first perturbation, (**b**) the second perturbation.

Although the posterior results for the parameters driving the term $\min\{\max\left\{\omega_{t},c\right\},d\}$ (i.e., ρ, ν, *p*, and obviously, *c* and *d* ) are very sensitive to the prior assumptions about parameter *c*, among others, the posterior inference about the conditional standard deviations (of the original and perturbed data, $\sigma_{t}$) are strikingly similar in terms of their dynamics (the posterior mean series are highly correlated); see Figure 23. However, in the LLSF-SV model with c∼INK(0.4;0.009), considerably smaller values of the posterior means of $\sigma_{t}$ are noted for the zero returns, although it can be noticed in the figure only at high magnification. The average value of the posterior means of $\sigma_{t}$ corresponding to the zero returns is equal to: 0.15 (with standard deviation 0.05) in the case of c∼INK(0.4;0.009), 1.00 (0.44) in the case of c∼INK(2;0.1), and 1.67 (0.69) in the t-SV model with ν∼G(8;0.8). The zero returns in the original data and the observations that are concentrated around the mode in the perturbed data (marked by the blue vertical lines in Figure 23, Figure 24 and Figure 25) tend to be treated in a special way by assigning to them relatively small values of $\omega_{t}$; see Figure 24 and Figure 25. Thus, in this sense, the LLFT-SV model is able to detect “inliers”, defined here as the observations that lie in the central part of empirical distribution and their occurrence is “atypical” in the sense of their repeatedness or their “atypically” high concentration around the mode.

Note that the inference about the mixing variables, $\omega_{t}$s, depends on the size of perturbation. It seems that the closer a point observation ($y_{t}$) is to zero (or to the mode), the smaller value of corresponding $\min\{\max\left\{\omega_{t},c\right\},d\}$ in the LLFT-SV model with c∼INK(0.4;0.009). Thanks to this prior assumption, $\min\{\max\left\{\omega_{t},c\right\},d\}$ can take on values close to zero and, in consequence, atypical “inlying” observations can be better explained. This result is in accordance with those of the predictive power comparison of models, presented in the previous subsection. Allowing *c* to take on low values considerably improves the predictive performance of the LLFT-SV model, due to its far more appropriate handling of the very center of the data distribution’s central part.

### 4.3. Results for Stock Market Indices

**Figure 26:**
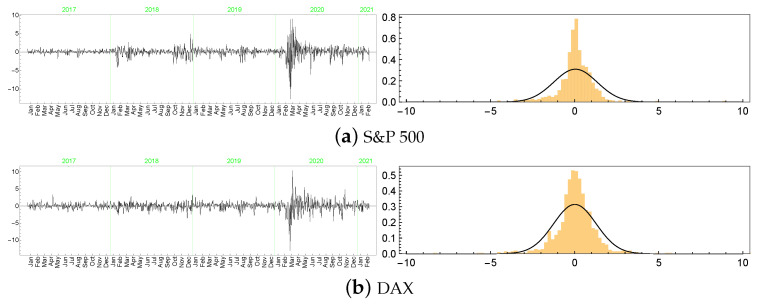
The series (left) and histograms (with fitted Gaussian curves; right) of the S&P 500 and DAX returns.

In this section, we present only a brief analysis of the LLFT-SV model estimation results for two common stock market indices: Standard and Poor’s 500 (S&P 500) and Deutscher Aktienindex (DAX), both representing “typical” financial series, with their dynamics reflecting movements of the entire market rather than only of a single company’s stock prices (possibly, of a limited liquidity). Both data sets were downloaded from http://stooq.pl (an open access web page) and cover the same period as the one considered previously for MSAG.DE: 28 December 2016 through 25 February 2021. As seen in Figure 26, the logarithmic rates of return reveal strong leptokurticity and fat tails, with the latter built up particularly due to the market volatility outburst triggered by the COVID-19 pandemic. However, contrary to the MSAG.DE data set, the series currently at hand do not feature zero returns nor any repeating value, therefore representing quite distinct characteristics.

Similarly to the analysis for MSAG.DE, the first two available observations are spared for the initial condition in the AR(2) structure, yielding the final number of modelled observation of T=1047 for DAX, and T=1044 for S&P 500.

**Figure 27:**
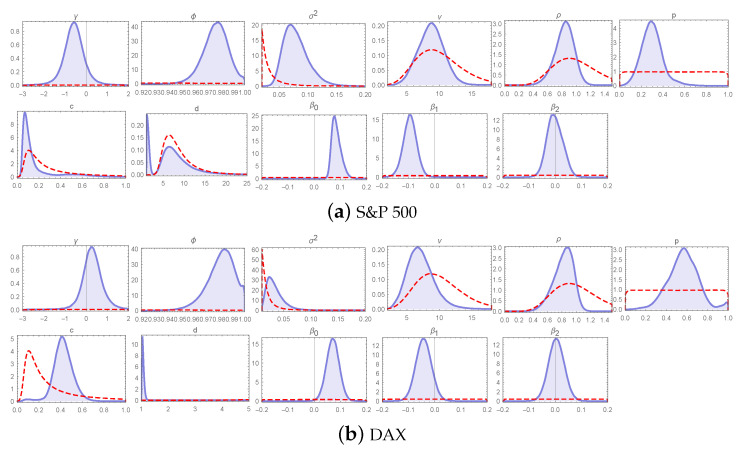
Marginal posteriors (blue line) and priors (red line) of the LLFT-SV model parameters under ν∼G(8;0.8), c∼INK(0.4;0.009) and d∼INK(2;100). The top two rows show plots for S&P 500 and the bottom two rows for DAX.

Posterior characteristics of the key parameters of the LLFT-SV model (ν, ρ, *p*) indicate the validity of modelling the error term through the LLFT distribution, pointing to a non-negligible contribution of both mixture components: beta and Pareto (in the SMN representation); see Table 7. As implied by the posterior expectations of *p*, for DAX, both components are almost of the same weight, while in the case of S&P 500, the beta component constitutes roughly 30% of the mixture. Higher posterior medians of *c* for DAX, as compared to S&P 500, indicate a lower local conditional peakedness, which is suitably accompanied by lower posterior medians of *d*, the values of which, being close to 1, bring the conditional distribution of $\varepsilon_{t}$ (given *d*) closer to a normal distribution. However, it seems that in the case of S&P 500 the posterior distributions of *c* and *d* are visibly affected by the priors (see relevant panels in Figure 27).

In general, we could conclude that incorporating the LLFT distribution into an SV model may be an empirically valid extension of the basic stochastic volatility structure also for “more typical” financial time series, such as market indices, rather than only for some specific company’s (“less typical”) stock returns.

## 5. Discussion

In the paper, we proposed a new scale mixture of normal distributions that features local leptokurticity and local fat tails. This new LLFT distribution was further incorporated into a basic stochastic volatility model (yielding a LLFT-SV specification), so as to enhance the model’s capability of capturing corresponding empirical properties of financial time series. For the new model, we developed a Bayesian framework along with valid MCMC methods of posterior sampling. Empirical results indicate the validity of the LLFT-SV specification for modelling both “non-standard” financial time series with repeating zero returns (resulting in a single pronounced histogram bar), as well as more “typical” data on the S&P 500 and DAX indices. For the former, the LLFT-SV model was also shown to markedly outperform a common, globally heavy-tailed, t-SV alternative in terms of density forecasting (as measured by the sum of predictive likelihoods). Apparently, the noticeable predictive superiority of the LLFT-SV model draws on its more adequate handling of the peak in the central part of the returns’ distribution but not at the expense of a valid modelling of the outliers.

In general, it can be concluded that it is not only fat tails but also close-to-mode returns that are vital to financial data modelling. Thus, more flexible than common heavy-tailed (such as Student’s *t* or slash) distributions may be empirically required so that both features can be ’freely’ controlled for by separate parameters (rather than a single one). Nonetheless, the LLFT-SV specification proposed in this study leaves some important room for further improvement, with the model’s most obvious limitations being the symmetry of the conditional distribution and the preclusion of the leverage effect. The symmetry of the LLFT distribution suggests that it lends itself mainly to modelling either time series with quite a symmetric data distribution or at least those where an adequate capturing of the tails and peakedness would empirically prove more important than allowing for skewness (although this could not be settled without comparing alternative models). Obviously, extending the LLFT-SV model towards the asymmetry could be directly related to it gaining the ability of accounting for the leverage effect. Moreover, skewing the LLFT distribution itself seems a valid and important extension. We leave such generalizations of our current framework for future work. Finally, and on a different note, evaluation of the LLFT-SV model in terms of risk modelling and management would also be most desirable.

## Figures and Tables

**Table 1:** Sample characteristics for the MSAG.DE data set.

Mean	St. Dev.	Median	Min	Max	Skewness	Kurtosis	Percent of Zero Returns
−0.035	2.851	0.000	−22.399	30.390	0.635	24.780	11%

**Table 2:** Posterior characteristics of model parameters for the original MSAG.DE data set. The first row of each entry: posterior median. The second row: 90% posterior confidence interval (in parentheses). The third row: interquartile range.

Parameter	LLFT-SV	LLFT-SV	LLFT-SV	t-SV	t-SV
	ν∼G(0.2;0.05)	ν∼G(8;0.8)	ν∼G(8;0.8)	ν∼G(0.2;0.05)	ν∼G(8;0.8)
	c∼INK(2;0.1)	c∼INK(2;0.1)	c∼INK(2;0.1)	**–**	**–**
	d∼INK(9;100)	d∼INK(9;100)	d∼INK(2;100)	**–**	**–**
γ	0.947	1.290	1.206	0.982	1.045
	(0.492, 1.500)	(0.772, 1.682)	(0.758, 1.661)	(0.709, 1.255)	(0.779, 1.304)
	0.406	0.413	0.392	0.224	0.210
ϕ	0.914	0.905	0.911	0.905	0.899
	(0.860, 0.950)	(0.854, 0.944)	(0.860, 0.947)	(0.851, 0.947)	(0.839, 0.941)
	0.036	0.036	0.036	0.036	0.042
$\sigma^{2}$	0.150	0.171	0.156	0.156	0.186
	(0.078, 0.261)	(0.099, 0.282)	(0.087, 0.270)	(0.087, 0.282)	(0.102, 0.324)
	0.072	0.072	0.069	0.078	0.087
ν	3.99	6.30	5.60	5.11	6.44
	(2.73, 7.35)	(3.64, 8.82)	(3.57, 8.96)	(3.64, 8.54)	(4.34, 10.85)
	1.82	2.38	2.24	1.68	2.52
ρ	0.64	0.67	0.66	–	–
	(0.22, 0.98)	(0.24, 0.97)	(0.23, 0.96)	–	–
	0.33	0.32	0.35	–	–
*p*	0.148	0.240	0.214	–	–
	(0.046, 0.302)	(0.106, 0.363)	(0.107, 0.382)	–	–
	0.087	0.125	0.131	–	–
*c*	0.134	0.120	0.118	–	–
	(0.061, 0.261)	(0.058, 0.231)	(0.057, 0.220)	–	–
	0.077	0.067	0.067	–	–
*d*	5.161	3.354	7.553	–	–
	(1.144, 10.673)	(1.105, 6.396)	(1.105, 16.632)	–	–
	2.951	3.146	4.524	–	–
$\beta_{0}$	−0.043	−0.040	−0.037	−0.076	−0.082
	(−0.130, 0.008)	(−0.121, 0.008)	(−0.118, 0.008)	(−0.163, 0.008)	(−0.166, 0.005)
	0.066	0.063	0.060	0.069	0.069
$\beta_{1}$	−0.046	-0.037	−0.034	−0.115	−0.118
	(−0.118, 0.005)	(−0.100, 0.005)	(−0.097, 0.005)	(−0.163, −0.067)	(−0.166, −0.067)
	0.060	0.048	0.051	0.042	0.039
$\beta_{2}$	0.023	0.023	0.023	0.035	0.035
	(−0.004, 0.065)	(−0.001, 0.065)	(−0.001, 0.062)	(−0.010, 0.080)	(−0.010, 0.083)
	0.030	0.030	0.027	0.036	0.039

**Table 3:** Basic characteristics (averages, standard deviations, correlation coefficients) of the posterior means of latent processes, in models with ν∼G(8;0.8), c∼INK(2;0.1), and d∼INK(2;100).

Latent Process	Model Type	Average	Standard Deviation	Correlation Coefficient
$h_{t}$	LLFT-SV	5.865	9.126	0.994
	t-SV	4.854	6.710	
$\min\{\max\left\{\omega_{t},c\right\},d\}$	LLFT-SV	1.071	0.234	0.759
$\sqrt{\omega_{t}}$	t-SV	1.148	0.154	
$\sqrt{h_{t}}\min\left\{\max\left\{\omega_{t},c\right\},d\right\}$	LLFT-SV	2.119	1.051	0.964
$\sqrt{h_{t}\omega_{t}}$	t-SV	2.173	1.210	

**Table 4:** Sums of the log predictive likelihoods.

Forecast	LLFT-SV	LLFT-SV	t-SV
Horizon	(c=0.118,d=7.553)	(c=0.014,d=2.808)	
h=1	−261.66	−239.82	−263.84
h=2	−264.43	−241.64	−266.59
h=3	−264.48	−241.30	−266.15
h=4	−266.56	−245.16	−267.87
h=5	−267.93	−246.56	−269.02
h=6	−264.99	−242.72	−266.61
h=7	−265.45	−243.37	−267.13
h=8	−265.57	−242.87	−267.14
h=9	−265.01	−241.98	−266.73
h=10	−267.47	−244.50	−269.49

**Table 5:** Posterior characteristics of model parameters for the perturbed and original MSAG.DE data, under c∼INK(2;0.1). The first row of each entry: posterior median. The second row: 90% posterior confidence interval (in parentheses). The third row: interquartile range.

Parameter	Original Data	Perturbed Data	Original Data	Perturbed Data
	LLFT-SV	LLFT-SV	t-SV	t-SV
		$U(-5\times 10^{-4},5\times 10^{-4})$		$U(-5\times 10^{-4},5\times 10^{-4})$
	ν∼G(8;0.8)	ν∼G(8;0.8)	ν∼G(8;0.8)	ν∼G(8;0.8)
	c∼INK(2;0.1)	c∼INK(2;0.1)	**–**	**–**
	d∼INK(2;100)	d∼INK(2;100)	**–**	**–**
γ	1.206	1.227	1.045	1.045
	(0.758, 1.661)	(0.800, 1.654)	(0.779, 1.304)	(0.786, 1.304)
	0.392	0.371	0.210	0.210
ϕ	0.911	0.914	0.899	0.902
	(0.860, 0.947)	(0.863, 0.950)	(0.839, 0.941)	(0.842, 0.944)
	0.036	0.033	0.042	0.039
$\sigma^{2}$	0.156	0.150	0.186	0.177
	(0.087, 0.270)	(0.084, 0.255)	(0.102, 0.324)	(0.096, 0.309)
	0.069	0.066	0.087	0.087
ν	5.60	5.67	6.44	6.44
	(3.57, 8.96)	(3.71, 8.89)	(4.34, 10.85)	(4.34, 10.71)
	2.24	2.17	2.52	2.45
ρ	0.66	0.70	–	–
	(0.23, 0.96)	(0.35, 0.97)	–	–
	0.35	0.26	–	–
*p*	0.214	0.226	–	–
	(0.107, 0.382)	(0.109, 0.381)	–	–
	0.131	0.126	–	–
*c*	0.118	0.127	–	–
	(0.057, 0.220)	(0.072, 0.229)	–	–
	0.067	0.057	–	–
*d*	7.553	7.462	–	–
	(1.105, 16.632)	(1.118, 16.376)	–	–
	4.524	4.472	–	–
$\beta_{0}$	−0.037	−0.049	−0.082	−0.079
	(−0.118, 0.008)	(−0.124, 0.005)	(−0.166, 0.005)	(−0.166, 0.005)
	0.060	0.054	0.069	0.069
$\beta_{1}$	−0.034	−0.043	−0.118	−0.118
	(−0.097, 0.005)	(−0.100, −0.001)	(−0.166, −0.067)	(−0.166, −0.067)
	0.051	0.042	0.039	0.039
$\beta_{2}$	0.023	0.029	0.035	0.038
	(−0.001, 0.062)	(−0.001, 0.065)	(−0.010, 0.083)	(−0.010, 0.086)
	0.027	0.027	0.039	0.036

**Table 6:** Posterior characteristics of the LLFT-SV model parameters for the perturbed and original MSAG.DE data, under c∼INK(0.4;0.009). The first row of each entry: posterior median. The second row: 90% posterior confidence interval (in parentheses). The third row: interquartile range.

Parameter	Perturbed Data	Perturbed Data	Original Data
	$U(-5\times 10^{-4},5\times 10^{-4})$	$U(-5\times 10^{-5},5\times 10^{-5})$	
	c∼INK(0.4;0.009)	c∼INK(0.4;0.009)	c∼INK(0.4;0.009)
	d∼INK(2;100)	d∼INK(2;100)	d∼INK(2;100)
γ	0.800	−0.005	−0.047
	(0.408, 1.129)	(−0.278, 0.387)	(−0.306, 0.219)
	0.273	0.231	0.211
ϕ	0.929	0.917	0.911
	(0.881, 0.962)	(0.866, 0.950)	(0.860, 0.947)
	0.033	0.033	0.033
$\sigma^{2}$	0.102	0.135	0.153
	(0.048, 0.183)	(0.081, 0.225)	(0.090, 0.255)
	0.054	0.057	0.066
ν	3.36	1.040	1.032
	(1.47, 4.83)	(1.003, 2.394)	(1.002, 1.133)
	0.98	0.07	0.05
ρ	0.11	0.047	0.046
	(0.06, 0.17)	(0.027, 0.079)	(0.029, 0.078)
	0.04	0.02	0.020
*p*	0.119	0.1170	0.117
	(0.097, 0.144)	(0.100, 0.136)	(0.100, 0.136)
	0.019	0.015	0.015
*c*	0.021	0.014	0.014
	(0.018, 0.025)	(0.012, 0.015)	(0.012, 0.016)
	0.002	0.001	0.001
*d*	9.503	2.795	2.806
	(2.316, 18.985)	(2.496, 10.764)	(2.514, 3.496)
	4.706	0.260	0.230
$\beta_{0}$	−0.002	−0.0001	−0.0001
	(−0.007, 0.003)	(−0.0017, 0.001)	(−0.001, 0.002)
	0.003	0.001	0.001
$\beta_{1}$	−0.0003	−0.0002	0.0000
	(−0.003, 0.003)	(−0.0013, 0.0008)	(−0.0004, 0.001)
	0.0025	0.0005	0.001
$\beta_{2}$	0.0015	0.0000	0.0000
	(−0.001, 0.0044)	(−0.0009, 0.0011)	(−0.0004, 0.001)
	0.002	0.0008	0.0007

**Table 7:** Posterior characteristics of the LLFT-SV model parameters for DAX and S&P 500, under d∼INK(2;100). The first row of each entry: posterior median. The second row: 90% posterior confidence interval (in parentheses). The third row: interquartile range.

Parameter	DAX	DAX	S&P 500	S&P 500
	ν∼G(8;0.8)	ν∼G(8;0.8)	ν∼G(8;0.8)	ν∼G(8;0.8)
	c∼INK(2;0.1)	c∼INK(0.4;0.009)	c∼INK(2;0.1)	c∼INK(0.4;0.009)
γ	0.191	0.296	−0.558	−572
	(−0.649, 1.080)	(−0.565, 1.269)	(−1.377, 0.289)	(−1.419, 0.303)
	0.560	0.588	0.581	0.588
ϕ	0.983	0.983	0.980	0.980
	(0.962, 0.998)	(0.962, 0.998)	(0.962, 0.995)	(0.962, 0.995)
	0.012	0.015	0.012	0.012
$\sigma^{2}$	0.033	0.033	0.081	0.078
	(0.018, 0.066)	(0.015, 0.063)	(0.054, 0.120)	(0.051, 0.123)
	0.021	0.018	0.027	0.027
ν	6.72	7.00	8.33	8.75
	(3.99, 10.15)	(4.13, 10.85)	(5.18, 11.48)	(5.53, 11.90)
	2.52	2.66	2.52	2.59
ρ	0.83	0.84	0.86	0.85
	(0.57, 1.02)	(0.59, 1.02)	(0.61, 1.05)	(0.60, 1.04)
	0.19	0.18	0.18	0.17
*p*	0.506	0.573	0.309	0.294
	(0.275, 0.718)	(0.332, 0.789)	(0.175, 0.476)	(0.147, 0.475)
	0.197	0.184	0.114	0.121
*c*	0.408	0.421	0.198	0.103
	(0.253, 0.556)	(0.294, 0.568)	(0.122, 0.586)	(0.051, 0.591)
	0.115	0.104	0.134	0.101
*d*	1.079	1.066	5.643	6.369
	(1.027, 6.851)	(1.027, 5.4866)	(1.089, 14.52)	(1.089, 15.147)
	0.065	0.052	7.326	7.821
$\beta_{0}$	0.071	0.071	0.095	0.083
	(0.032, 0.110)	(0.032, 0.113)	(0.068, 0.122)	(0.062, 0.119)
	0.030	0.033	0.021	0.024
$\beta_{1}$	−0.043	−0.040	−0.097	−0.094
	(−0.091, 0.005)	(−0.088, 0.008)	(−0.142, −0.049)	(−0.139, −0.052)
	0.039	0.039	0.036	0.033
$\beta_{2}$	0.008	0.005	−0.004	−0.001
	(−0.040, 0.059)	(−0.043, 0.056)	(−0.052, 0.044)	(−0.046, 0.050)
	0.042	0.042	0.036	0.042

**Figure A1:**
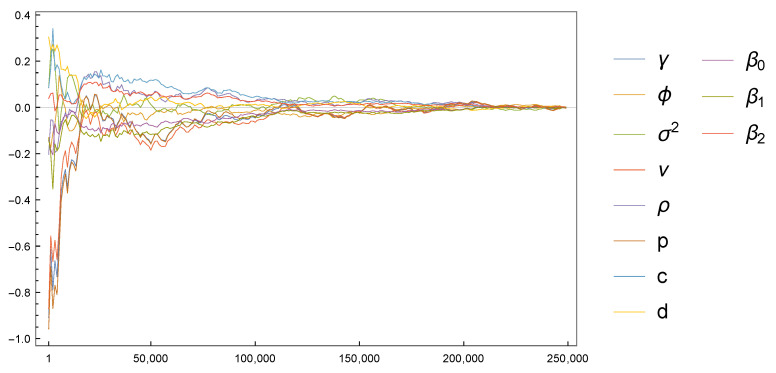
Standardized CUSUM for the parameters.

**Figure A2:**
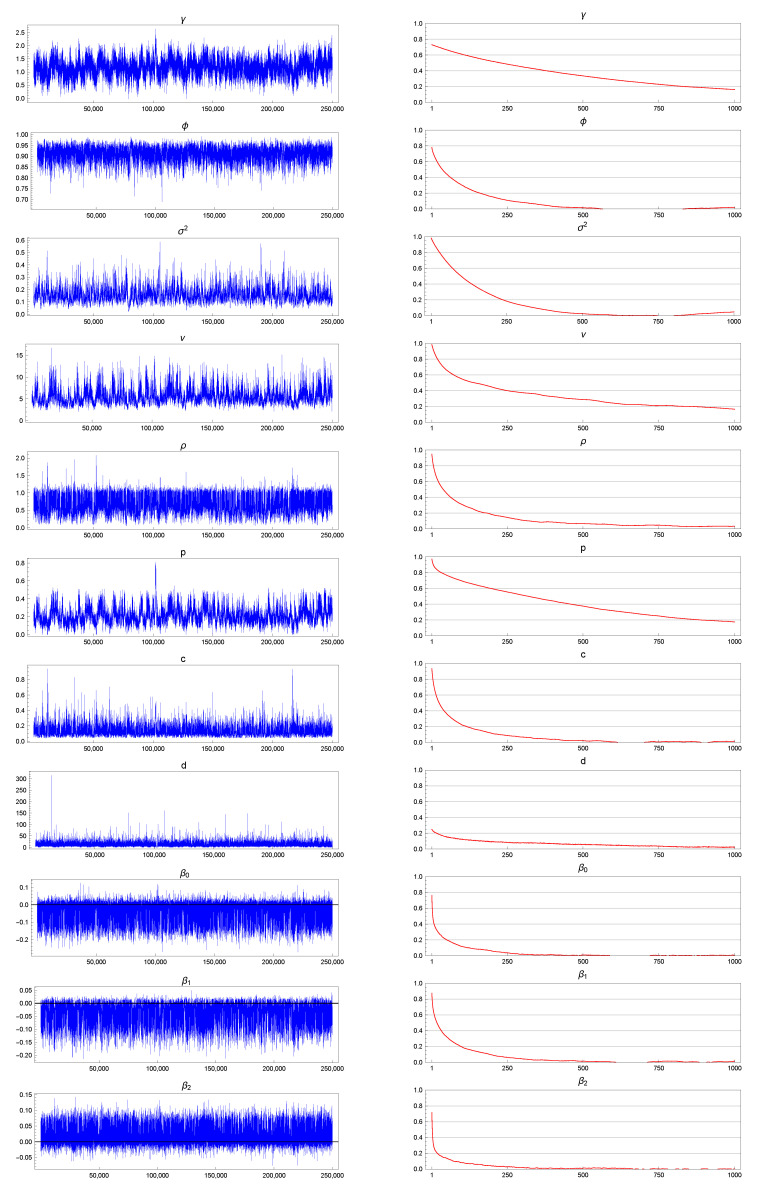
Trace plots and ACF for MCMC samples.

## Data Availability

The data are publicly available.

## Appendix A

In this appendix, we provide and theorems showing that the marginal data density value (i.e., marginal likelihood) is finished in the Bayesian LLFT-SV model considered in Section 3.

**Theorem** **A1.**
*For the stochastic volatility model presented in Section 3, and under the additional assumption that the inverse gamma prior distribution of $\sigma^{2}$ is truncated to the interval $\left(0,\kappa_{\sigma }^{2}\right)$, for any fixed $\kappa_{\sigma }^{2}<\infty$, we have that p(y)<∞.*

**Proof** **of** **Theorem** **2** Denote $p\left(\omega |p,\rho ,\nu \right)=\prod_{t=1}^{T}\left(p\;f_{Beta}\left(\omega_{t}|\rho ,\;1\right)+\left(1-p\right)f_{Pareto}\left(\omega_{t}|1,\nu \right)\right)$ and $\tilde{h}={\left[\ln h_{1},\ln h_{2},\ldots ,\ln h_{T}\right]}^{\prime }$. Note that

$$\begin{array}{c} p\left(y\right)=\int_{\omega }\int_{\tilde{h}}\int_{\beta }\int_{\sigma }\int_{\gamma }\int_{\phi }\int_{p}\int_{\nu }\int_{\rho }\int_{c}\int_{d}p\left(y,\omega ,\tilde{h},\beta ,\sigma ,\gamma ,\phi ,\;p,\nu ,\;\rho ,c,\;d\;|y_{\left(0\right)}\right)\\ dd\;dc\;d\rho \;d\nu \;dp\;d\phi \;d\gamma \;d\sigma \;d\beta \;d\tilde{h}\;d\omega \propto \\ \int_{\omega }\int_{\tilde{h}}\int_{\beta }\int_{\sigma }\int_{\gamma }\int_{\phi }\int_{p}\int_{\nu }\int_{\rho }\int_{c}\int_{d}\underset{f_{1}}{\underset{︸}{\frac{e^{-\frac{1}{2}\sum_{t=1}^{T}{\left(\frac{y_{t}-x_{t}\beta }{e^{{\tilde{h}}_{t}/2}\min\left\{\max\left\{\omega_{t},c\right\},d\right\}}\right)}^{2}}}{\prod_{t=1}^{T}\min\left\{\max\left\{\omega_{t},c\right\},d\right\}}}}\times \underset{f_{2}}{\underset{︸}{\frac{e^{-\frac{1}{2}\sum_{t=1}^{T}\left({\left(\frac{{\tilde{h}}_{t}-\gamma -\phi \left({\tilde{h}}_{t-1}-\gamma \right)}{\sigma }\right)}^{2}+{\tilde{h}}_{t}\right)}}{\sigma^{T}}}}\times \\ p\left(\omega |p,\rho ,\nu \right)p\left(\sigma \right)p\left(\beta \right)p\left(p\right)p\left(\;\nu \right)p\left(\rho \right)p\left(d\right)p\left(c\right)dd\;dc\;d\rho \;d\nu \;dp\;d\phi \;d\gamma \;d\sigma \;d\beta \;d\tilde{h}\;d\omega . \end{array}$$

For the term $f_{1}$, we have the following inequality $f_{1}\leq \frac{e^{-\frac{1}{2d^{2}}\sum_{t=1}^{T}{\left(\frac{y_{t}-x_{t}\beta }{e^{{\tilde{h}}_{t}/2}}\right)}^{2}}}{c^{T}}.$ Using this inequality, we get that there exist $C_{1},C_{2}<\infty$ such that

(A1) $$\begin{array}{c} \int_{c}\int_{d}f_{1}p\left(d\right)p\left(c\right)dd\;dc\leq C_{1}\left(\frac{1}{2}\sum_{t=1}^{T}e^{-{\tilde{h}}_{t}}\left(y_{t}-x_{t}\beta \right){}^{2}+\beta_{d}\right){}^{-\alpha_{d}}\times \\ \left(\Gamma \left(\alpha_{d}\right)-\Gamma \left(\alpha_{d},\beta_{d}+\frac{1}{2}\sum_{t=1}^{T}e^{-{\tilde{h}}_{t}}\left(y_{t}-x_{t}\beta \right){}^{2}\right)\right)\leq C_{2} \end{array}$$

The term $f_{2}$ is the kernel of a multivariate Gaussian distribution for $\tilde{h}$, which can be written as

(A2) $$f_{2}={\left|\Sigma_{h}\right|}^{\frac{1}{2}}e^{-\frac{1}{2}{\left(\tilde{h}-r\right)}^{\prime }\Sigma_{h}\left(\tilde{h}-r\right)}e^{-\frac{\gamma }{2}\left(T-\sum_{t=1}^{T}\phi^{t}\right)}e^{-h_{0}\frac{\phi \left(\phi^{T}-1\right)}{\phi -1}}e^{\sigma^{2}g\left(\phi \right)},$$

where $\Sigma_{h}={\left[a_{ij}\right]}_{T\times T}$ is a tridiagonal matrix such that $|\Sigma_{h}{|}^{\frac{1}{2}}=\sigma^{-T}$, with the elements on the main diagonal: $a_{ii}=\frac{\phi^{2}+1}{\sigma^{2}}$, for i=1,2,…,T−1, $a_{TT}=\frac{1}{\sigma^{2}}$ and $a_{ij}=-\frac{\phi }{\sigma^{2}}$, for |i−j|=1, and r is the mean vector. The term g(ϕ) is a polynomial of order T−2 with positive values for ϕ∈(−1,1) and with known coefficients that depend only on the sample’s size, *T*. Hence, there exists $C_{3}<\infty$, which depends only on *T*, and such that $g\left(\phi \right)\leq C_{3}$. Using (A2), (A3) and inequalities $e^{-h_{0}\frac{\phi \left(\phi^{T}-1\right)}{\phi -1}}\leq e^{|h_{0}|T}$, $e^{-\frac{\gamma }{2}\left(T-\sum_{t=1}^{T}\phi^{t}\right)}\leq e^{|\gamma |T}$, $e^{\kappa_{\sigma }^{2}g\left(\phi \right)}\leq e^{\kappa_{\sigma }^{2}C_{3}}$, and integrating over $\tilde{h}$, we get that there exists $C_{4}<\infty$ such that

$$\begin{array}{c} p\left(y\right)\leq C_{4}\int_{\omega }\int_{\beta }\int_{\sigma }\int_{\gamma }\int_{\phi }\int_{p}\int_{\nu }\int_{\rho }e^{|\gamma |T}\times \\ p\left(\omega |p,\rho ,\nu \right)p\left(\sigma \right)p\left(\beta \right)p\left(p\right)p\left(\;\nu \right)p\left(\rho \right)d\rho \;d\nu \;dp\;d\phi \;d\gamma \;d\sigma \;d\beta \;d\omega =C_{4}\int_{\gamma }e^{|\gamma |T}p\left(\gamma \right)d\gamma <\infty \end{array}$$

which ends the proof. □

## Appendix B

We examine the convergence of the MCMC sampler through a visual inspection of ACF, standardized CUSUM ([36,37,38]) and trace plots; see Figure A1 and Figure A2. Furthermore, in Table A1, inefficiency factors are reported (calculated with the batch-means method, using the ACF lags from 1 through 500; see e.g., [39]).

The results presented in Figure A1 and Figure A2 do not display any sort of anomalies that would invalidate the MCMC convergence for the MSAG.DE data (plots obtained for the S&P 500 and DAX indices are similar, and therefore, for saving the space, not reported here, although available upon request). The ACF plots reveal, however, a typical autocorrelation, most persistent for parameters γ, ν and *p*, which is further reflected in the inefficiency factors (see Table A1).

**Table A1:** Inefficiency factors obtained in the LLFT-SV model, under ν∼IG(8;0.8), d∼INK(2;100), c∼INK(2;0.1).

Parameter	MSAG.DE	DAX	S&P 500
γ	16.743	6.197	2.553
ϕ	11.507	13.184	7.219
$\sigma^{2}$	14.419	17.611	12.23
ν	16.275	11.676	10.114
ρ	11.997	8.651	4.837
*p*	18.022	21.078	13.499
*c*	10.688	12.954	15.081
*d*	7.696	15.471	8.127
$\beta_{0}$	7.654	4.012	5.363
$\beta_{1}$	9.752	3.137	2.985
$\beta_{2}$	6.468	5.554	2.858
